# Mechanism of salidroside promoting testosterone secretion induced by H_2_O_2_ in TM3 Leydig cells based on metabolomics and network pharmacology

**DOI:** 10.3389/fchem.2025.1544876

**Published:** 2025-02-27

**Authors:** Zixu Wang, Yunlong Xu, Huazhong Xiong

**Affiliations:** ^1^ College of Traditional Chinese Medicine, Changchun University of Chinese Medicine, Changchun, China; ^2^ Prevention and Treatment Center, Affiliated Hospital to Changchun University of Chinese Medicine, Changchun, China

**Keywords:** salidroside, oxidative stress, testosterone, metabolomics, network pharmacology

## Abstract

Oxidative stress-induced damage is a significant contributor to the impairment of Leydig cells in the testes, potentially diminishing the secretion of testosterone and other androgens, thereby resulting in testosterone deficiency. Salidroside, the principal bioactive constituent derived from Rhodiola, exhibits potent antioxidant properties. This study aims to investigate the underlying mechanisms by which salidroside enhances testosterone secretion. The study investigated the oxidative damage in TM3 cells induced by H_2_O_2_ and demonstrated that salidroside significantly decreased the levels of ROS and MDA, while increasing the levels of testosterone, SOD, GSH. These changes effectively ameliorated oxidative stress, mitigated oxidative damage, protected TM3 cells, and enhanced testosterone secretion. Additionally, UPLC-QE-Orbitrap-MS was employed to analyze the metabolomics of TM3 cells, identifying 28 distinct metabolites and associated metabolic pathways. Key metabolic pathways identified include Arginine biosynthesis, Alanine, aspartate and glutamate metabolism, Citrate cycle (TCA cycle), Phenylalanine metabolism, Pyruvate metabolism. Utilizing network pharmacology, the core targets of salidroside in enhancing testosterone secretion were further investigated, revealing the involvement of AMACR, CYP3A4, ECHS1, HSD17B10, MPO, and TYR. This discovery was confirmed by dry-wet analysis. To sum up, salidroside can reduce the level of oxidative stress and promote testosterone secretion through multiple metabolic pathways and multiple targets. In a word, salidroside may provide a new strategy for preventing and treating testosterone deficiency.

## 1 Introduction

Testosterone (T) is the predominant androgen secreted in males, playing a crucial role in physiological functions. Inadequate secretion of testosterone can result in conditions such as erectile dysfunction, fatigue, and osteoporosis (Aversa and Morgentaler, 2015). Testicular interstitial cells, which are vital germ cells for sustaining normal reproductive physiology in male animals, are responsible for the synthesis and secretion of testosterone (Zhang et al., 2024). Research has demonstrated that oxidative stress-induced injury is a critical factor contributing to the damage of Leydig cells in the testes, leading to a reduction in the secretion of testosterone and other androgens, thereby adversely affecting spermatogenesis (Zhang et al., 2022). Consequently, mitigating oxidative stress in compromised cells and safeguarding the functionality of oxidatively damaged Leydig cells are of paramount importance for enhancing testosterone production (Dalmasso et al., 2017). The exogenous testosterone supplement therapy not only has safety problems, but also may inhibit its own testosterone secretion after taking it for a long time. Therefore, it has become a hot research topic to find effective components from natural drugs that can promote endogenous testosterone synthesis.

Salidroside, a principal bioactive compound in the traditional Chinese medicinal herb Rhodiola, exhibits notable antioxidant (Zhu and Yu, 2024), anti-inflammatory (Rasmussen et al., 2024), anti-apoptotic (Gao et al., 2023), and anti-aging (Li M. et al., 2024) properties. Studies indicate that salidroside administration enhances erectile function in rats with bilateral cavernous nerve injury (Ye et al., 2021). This improvement is attributed to its capacity to inhibit cellular apoptosis and fibrosis through the promotion of protective autophagy, as well as its ability to ameliorate the depletion of neural components, endothelial cells, and smooth muscle cells within the cavernous body. Furthermore, a recent study indicates that salidroside may enhance the Nrf2/HO-1 signaling pathway and improve erectile function in diabetic male rats by mitigating oxidative stress and apoptosis in cavernous tissue (Li Z. et al., 2024). While the protective effects of salidroside in various diseases have been highlighted, its potential role in promoting testosterone secretion requires further elucidation through more comprehensive research. Therefore, it is urgent to further study the exact mechanism of salidroside promoting testosterone secretion by improving oxidative stress, so as to promote its subsequent development and utilization.

In recent years, metabolomics, along with other omics techniques, has been extensively utilized in biological research (Maniscalco et al., 2020). Metabolomics is a research methodology that emulates the investigative approaches of genomics and proteomics, facilitating the quantitative analysis of all metabolites within organisms and identifying correlations between these metabolites and physiological or pathological changes (Pognan et al., 2023). Moreover, network pharmacology research grounded in metabolomics can integrate clustering algorithms and network topology to investigate the interrelationships among components, targets, and metabolites, thereby enabling the elucidation of underlying biological network mechanisms.

In this study, the differential metabolites and related metabolic pathways of oxidative damage of salidroside on TM3 cells were analyzed by metabolomics. Combined with network pharmacology, the metabolic pathways of differential metabolites were annotated by genes, and the regulatory mechanism of salidroside in improving oxidative damage of Leydig cells was revealed from the perspective of genes and metabolites, which laid the foundation.

## 2 Materials and methods

### 2.1 Material

#### 2.1.1 Materials and reagents

Salidroside was purchased from Shanghai Yuanye Technology Co., Ltd. (China, Shanghai), and the purity was over 98%. 30%H_2_O_2_ was purchased from Shanghai Macklin Biochemical Technology Co., Ltd. (China, Shanghai); DCFH-DA fluorescent probe was purchased from Beijing Solaibao Technology Co., Ltd. (China, Beijing). α-MEM medium, fetal bovine serum, penicillin (100 U/mL) and Streptomycin (100 μg/mL) were purchased from GIBCO Company. Testosterone, MDA, SOD and GSH kits were all purchased from Enzyme Immunity Biotechnology Co., Ltd. (China, Shanghai).

#### 2.1.2 Experimental cells

Mouse Leydig cells TM3 were purchased from the cell bank of China Academy of Sciences (China, Shanghai).

### 2.2 Cell culture

TM3 cells were maintained in DMEM/F12 medium supplemented with 10% fetal bovine serum, 100 U/mL penicillin, and 100 μg/mL streptomycin, incubated at 37°C with 5% CO_2_, and subcultured every 2–3 days.

### 2.3 Determination of TM3 cell viability by CCK-8 method

TM3 cells in the logarithmic growth phase were seeded into a 96-well plate at a concentration of 1 × 10^4^ cells per well, cultured for 24 h, and then the culture medium was discarded. 200 μL salidroside (1,5,10,20,50,100 μM) with different concentrations was added and cultured for 24 h, or H_2_O_2_ (1,10,50,100,200,400 μM) with different concentrations was cultured for 1 h. After adding 10 μL of CCK-8 solution to each well, the absorbance was measured at 450 nm following a 1-h incubation. Cell viability (%) = (OD _experimental groups_−OD _blank groups_)/ (OD _control groups_−OD _blank groups_) ×100%.

### 2.4 Effect of salidroside on the viability of TM3 cells induced by H_2_O_2_

Based on the method in “2.3,” they were grouped into control, H_2_O_2_-treated, and H_2_O_2_+Salidroside-treated group to investigate the impact of salidroside on the viability of TM3 cells induced by H_2_O_2_.

### 2.5 Detection of ROS content with DFCH-DA fluorescent probe

TM3 cells in logarithmic growth phase were inoculated in 6-well plates at a density of 1 × 10^6^/well for 24 h. The cells were subjected to 100 μM H_2_O_2_ for 1 h and treated with drugs in groups for 24 h. They were washed with PBS, stained with 10 μM DCFH-DA for 20 min in the dark, washed three times with PBS. ROS fluorescence probe DCFH-DA has excitation wavelength of 488 nm and emission wavelength of 525 nm, and the intracellular ROS level is detected by fluorescence microscope.

### 2.6 Enzyme-linked immunosorbent assay

Following the method in“2.3,” they were categorized into control, H_2_O_2_-treated, and H_2_O_2_+Salidroside-treated group. The cell supernatant was gathered after 24 h of culture. Following the ELISA kit guidelines, the levels of testosterone, MDA, SOD, and GSH in the cell culture solution were measured.

### 2.7 Metabonomics analysis

#### 2.7.1 Sample preparation of cell metabolomics

TM3 cells in logarithmic growth period were inoculated into culture bottles at a density of 1 × 10^7^/well and cultured for 24 h. Divided into control group, model group and treated group. 100 μM H_2_O_2_ was induced for 1 h and treated for 24 h. Add precooled PBS solution and wash twice, collect cells, centrifuge and discard supernatant. Quick frozen in liquid nitrogen for 15 min and stored at −80°C. The cells were repeatedly frozen and crushed for 5 times, added with 1 mL of methanol: water = 4:1 solution, vortex for 30 s, and ultrasonically crushed in ice bath for 6 min. Centrifuge at 4°C, 13,000 r/min for 10 min. Used for UPLC-QE-Orbitrap-MS analysis, the supernatant was transferred to the injection vial. Quality control (QC) samples were prepared by mixing equal volumes of supernatant samples.

#### 2.7.2 UPLC-QE-Orbitrap-MS analysis

AccucoreTMC18 column (2.1 × 100 mm, 1.7 µm). Mobile phase: 0.1% formic acid water (A) and acetonitrile (B), gradient elution: 0 ∼ 4 min, 98% ∼ 88% A; 4 ∼ 10 min, 88%∼76% A; 10–20 min, 76% ∼ 0% A; 20–28 min, 0% A. Flow rate: 0.3 mL/min. Sample volume: 10 μL. Column temperature: 25°C.

Ion source: electrospray ion source (ESI); Detection mode: positive ion and negative ion detection mode; FullMS resolution: 70,000; Dd-MS resolution: 17,500; Scanning range: m/z 66.7 ∼ 1,000; Atomizer temperature: 300°C; Drying gas temperature: 350°C; Capillary voltage: 3.5 kV; Breakage voltage: 220 V; Taper hole voltage: 65 V.

#### 2.7.3 Data processing

The original data undergo processing with Compound Discoverer software, which includes peak identification, extraction, alignment, and integration. Following standardization, the data underwent analysis using multivariate statistical methods such as principal component analysis (PCA) and orthogonal partial least squares discriminant analysis (OPLS-DA). Using VIP>1 and *p* < 0.05 as criteria, differential metabolites were screened and identified through the HMDB (https://www.hmdb.ca) and METLIN (https://metlin.scripps.edu). MetaboAnalyst 5.0 software (http://www.MetaboAnalyst.ca/) facilitated the analysis of metabolic pathways for the screened differential metabolites.

### 2.8 Network pharmacological analysis

#### 2.8.1 Analysis of the metabolic targets of diverse metabolites

The differential metabolites were input into the MetScape plug-in of Cytoscape 3.8.0 software to build a “differential metabolite-target” network.

#### 2.8.2 Disease and salidroside target acquisition

Through PubChem (https://pubchem.ncbi.nlm.nih.gov/), SwissTargetPrediction (http://www.swisstargetprediction.ch/), SuperPred (https://prediction.charity.de/subpages/target _ prediction. Screening and predicting the disease targets of “testosterone deficiency” with the help of GeneCards (https://www.genecards.org/), OMIM (https://www.omim.org/) and TTD (https://db.idrblab.net/ttd/) databases.

#### 2.8.3 PPI analysis

Identify salidroside, testosterone deficiency disorders, and metabolic targets, then use jvenn (https://jvenn.toulouse.inra.fr/app/example.html) to map interactive targets and determine the core targets. Targets related to the study were input into the String database (https://cn.string-db.org/cgi/input.pl) for analyzing protein-protein interactions, and visual analysis was conducted with Cytoscape 3.10.1 software.

#### 2.8.4 GO and KEGG analysis

The associated targets were entered into the Metascape database (https://metascape.org/gp/index.html#/main/step1) for enrichment analysis of GO and KEGG pathways, using a significance threshold of *p* < 0.05. The results were subsequently visualized utilizing the micro-information platform.

#### 2.8.5 Construction of “drug-metabolite-target-pathway-disease” network

The study identified signal pathways associated with testicular deficiency and sorted the genes in the top 20 pathways according to their P values. Finally, the drug-metabolite-target-pathway-disease network was constructed by Cytoscape 3.8.0 software.

### 2.9 Molecular docking

AutoDock Vina 1.2.2 was used to dock ligand components with receptor protein molecules, and the stability of ligand-receptor binding was analyzed according to the calculated binding energy. The binding mode of receptor and ligand was visualized by Discovery Studio 2019, which showed that ligand receptor was bound by hydrogen bonds and amino acid residues.

### 2.10 Molecular dynamics simulation

Utilizing the molecular docking results as the initial configuration, the system was simulated using Gromacs2022.2 software. The CHARMM36 force field was employed, and the TIP3P model was chosen for water representation. The system underwent solvation and ion equilibration processes. Subsequently, the canonical ensemble (NVT) and isothermal-isobaric ensemble (NPT) were applied to equilibrate the system, followed by conducting molecular dynamics simulations under standard temperature and pressure conditions. Following the molecular dynamics calculation, the structural features of the molecular dynamics trajectories were analyzed using root mean square deviation (RMSD), root mean square fluctuation (RMSF), gyration radius (RG), and the number of hydrogen bonds.

### 2.11 Detection of testosterone secretion related genes by RT-qPCR

TM3 cells were cultured at a density of 2 × 10^3^ cells per well and allocated into three groups: control, model, and salidroside treated. Following a 24 h treatment period, the cells were harvested for analysis. The total RNA was extracted by TRIzol method, and the cDNA was synthesized by reverse transcription with PrimeScript™ RT reagent Kit with gDNA Eraser (Perfect Real Time) kit. Real-time fluorescence quantitative PCR analysis was performed by TB Green^®^ Premix Ex Taq™ (Tli RNaseH Plus) kit to detect the expression of related genes. Using Primer-Blast tool of NCBI (https://www.ncbi.nlm.nih.gov/) database to query the mRNA sequence of target gene for primer design. Primer sequences are detailed in Table 1. GAPDH served as the internal reference gene for normalization, and the relative expression levels were calculated using the 2^−ΔΔCT^ method.

**TABLE 1:** RT-qPCR analysis of gene and associated basesequences.

GeneName	Forward (5′-3′)	Reverse (5′-3′)
GAPDH	CCT​CGT​CCC​GTA​GAC​AAA​ATG	TGA​GGT​CAA​TGA​AGG​GGT​CGT
CYP3A4	TGC​CTG​CTC​TTA​CTG​GCT​GGA​G	TGG​GTC​TGG​CTG​ACT​GGG​AAG
HSD17B10	GCA​TCC​AAA​GGG​GGC​ATA​GT	CTG​GCC​AAG​AAG​TTT​CGC​AC
MPO	CAG​TAG​ACC​CTC​GAA​TCG​CC	GGG​CCG​GTA​CTG​ATT​GTT​CA
TYR	ACA​CAC​TGG​AAG​TAT​TTT​TGA​ACA	TAG​GTG​CAT​TGG​CTT​CTG​GG
AMACR	TCA​CGA​TCC​AAC​GAA​AGG​CT	GCA​GAA​AGC​TGC​CAC​AAG​TT
ECHS1	TGG​GGA​TAA​GGC​CTT​TGC​AG	TGC​GAT​GAC​CGG​TTT​CTT​GA

### 2.12 Statistical analysis

All experiments should be repeated at least three times, and the values are expressed in mean ± SD. GraphPadPrism 8 software was used for statistical analysis. The statistical significance was determined by t-test or one-way analysis of variance (ANOVA). Among them, *p* < 0.05 was statistically significant.

## 3 Results

### 3.1 Effect of salidroside on oxidative damage of TM3 cells

**FIGURE 1:**
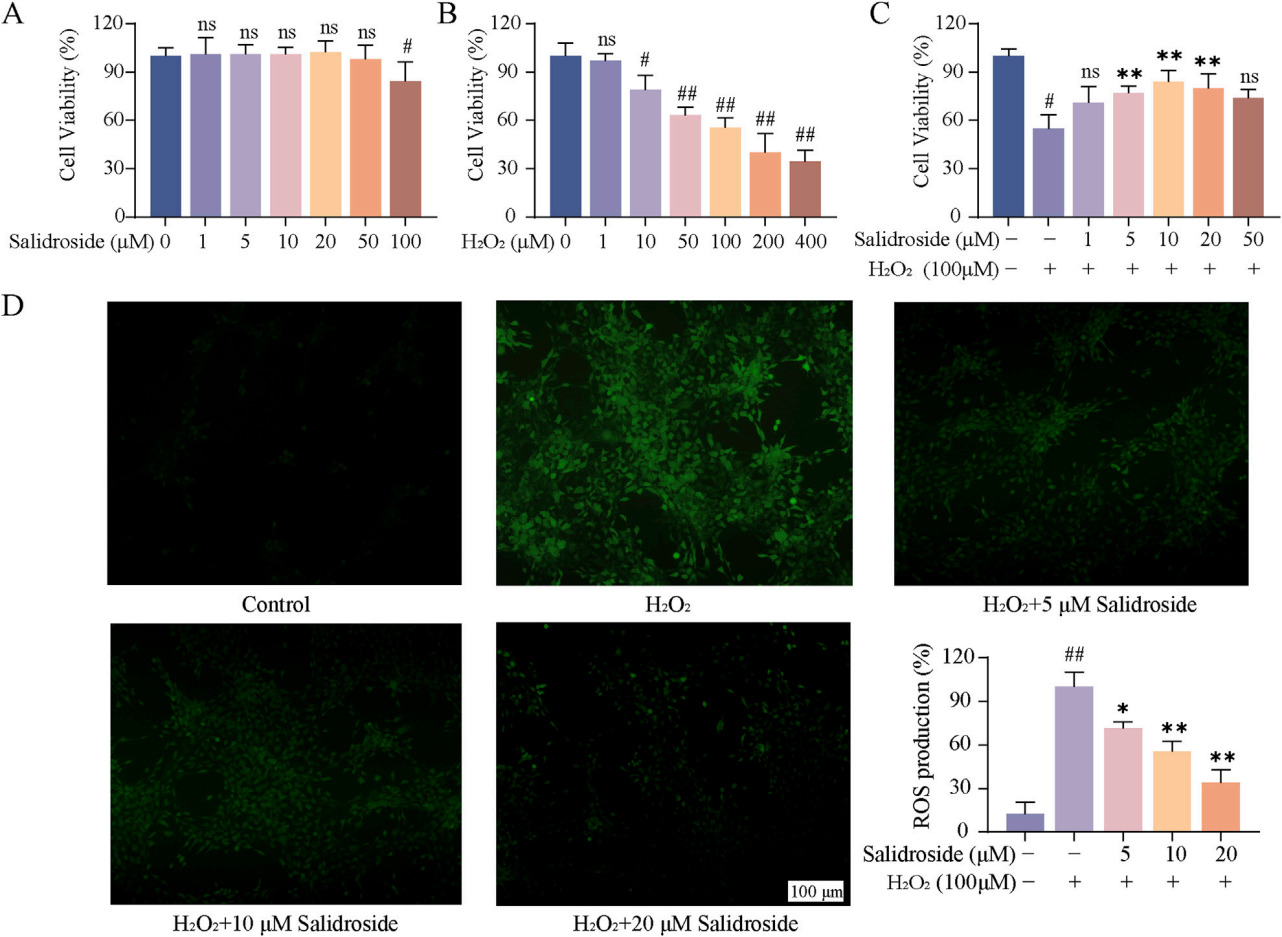
Effect of salidroside on oxidative damage of TM3 cells. **(A)** The effect of salidroside on the viability of TM3 cells. **(B)** Effect of H_2_O_2_ on the viability of TM3 cells. **(C)** Effect of salidroside on the viability of TM3 cells induced by H_2_O_2_. **(D)** The effect of salidroside on H_2_O_2_-induced ROS in TM3 cells. (mean ± SD, n = 3). Compared with the control group, ^#^ *p* < 0.05, ^##^ *p* < 0.01; Compared with the model group, *p < 0.05, **p < 0.01; Ns indicates that there is no significant difference.

As shown in Figure 1A, when the concentration of salidroside reached 100 μM, the cell viability decreased slightly. Therefore, 1, 5, 10, 20 and 50 μM were selected as the experimental concentrations for the following experiments. Figure 1B illustrates the induction of TM3 cells by varying concentrations of H_2_O_2_ over a 1 h period. It was observed that at H_2_O_2_ concentrations below 100 μM, the cell survival rate exceeded 50%. Conversely, when the concentration surpassed 200 μM, the cell survival rate fell below 50%, rendering it unsuitable for subsequent experimental operation. Consequently, a concentration of 100 μM H_2_O_2_ was selected to induce oxidative damage in TM3 cells. As depicted in Figure 1C, there was a statistically significant reduction in cell viability in the model group compared to the control group (*p* < 0.05). Compared with the model group, the cell viability of salidroside with different concentrations increased significantly (*p* < 0.05, *p* < 0.01). The result of ROS content is shown in Figure 1D. Compared with the control group, the relative content of ROS in TM3 cells in the model group increased (*p* < 0.01). Compared with the model group, the relative content of reactive oxygen species in salidroside group decreased (*p* < 0.05, *p* < 0.01).

### 3.2 Effects of salidroside on the contents of testosterone, SOD, MDA and GSH in TM3 cells

**FIGURE 2:**
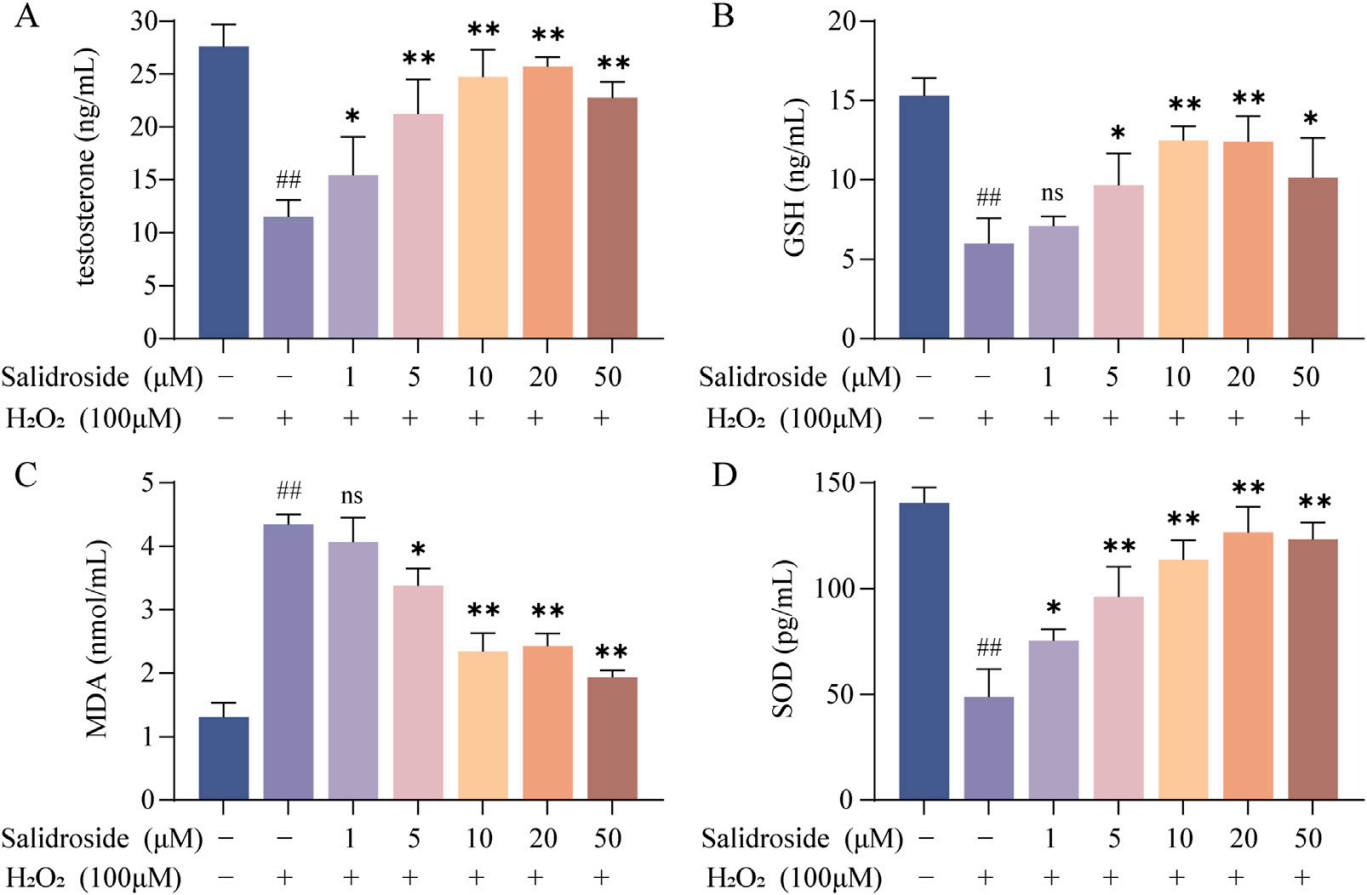
The contents of testosterone, SOD, MDA and GSH were detected by ELISA. **(A–D)** The effect of salidroside on the contents of testosterone, SOD, MDA and GSH in TM3 cells. (mean ± SD, n = 3). Compared with the control group, ^##^ *p* < 0.01; Compared with the model group, *p < 0.05, **p < 0.01; Ns indicates that there is no significant difference.

As shown in Figures 2A–D, the contents of testosterone, MDA, SOD and GSH in the cells of each group were compared, and the differences were statistically significant by analysis of variance (*p* < 0.05, *p* < 0.01). Compared with the control group, the level of MDA in TM3 cells in the model group increased, while the levels of testosterone, SOD and GSH decreased. Compared with the model group, the level of MDA in the salidroside group decreased, while the levels of testosterone, SOD and GSH increased in a concentration-dependent manner.

### 3.3 Metabolomics analysis

#### 3.3.1 Metabolomics QC sample detection

**FIGURE 3:**
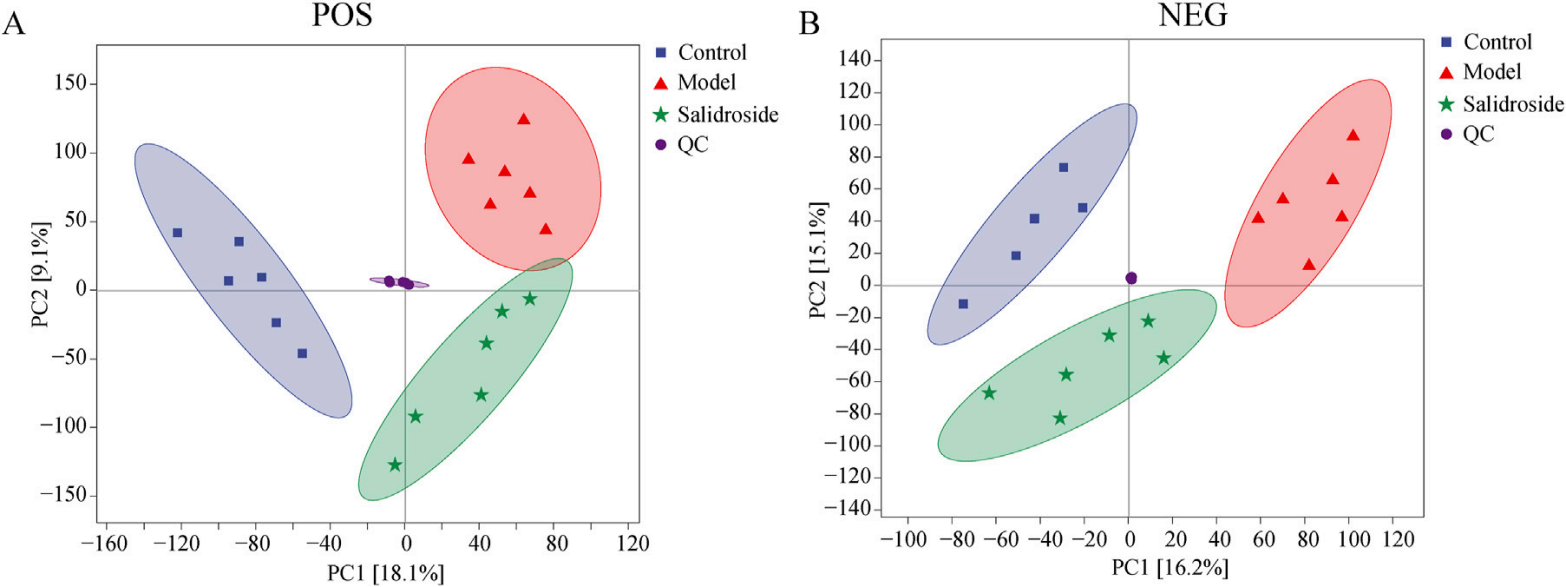
Score plots of QC, Sham, Model and salidroside groups in POS **(A)** and NEG mode **(B)**.

QC samples are evaluated during the analysis process to ensure the stability and reliability of the system and data. Then, all samples were analyzed by PCA. As can be seen in Figure 3, the QC samples show good clustering in the PCA score graph, demonstrating system and data stability.

#### 3.3.2 Multivariate statistical analysis

**FIGURE 4:**
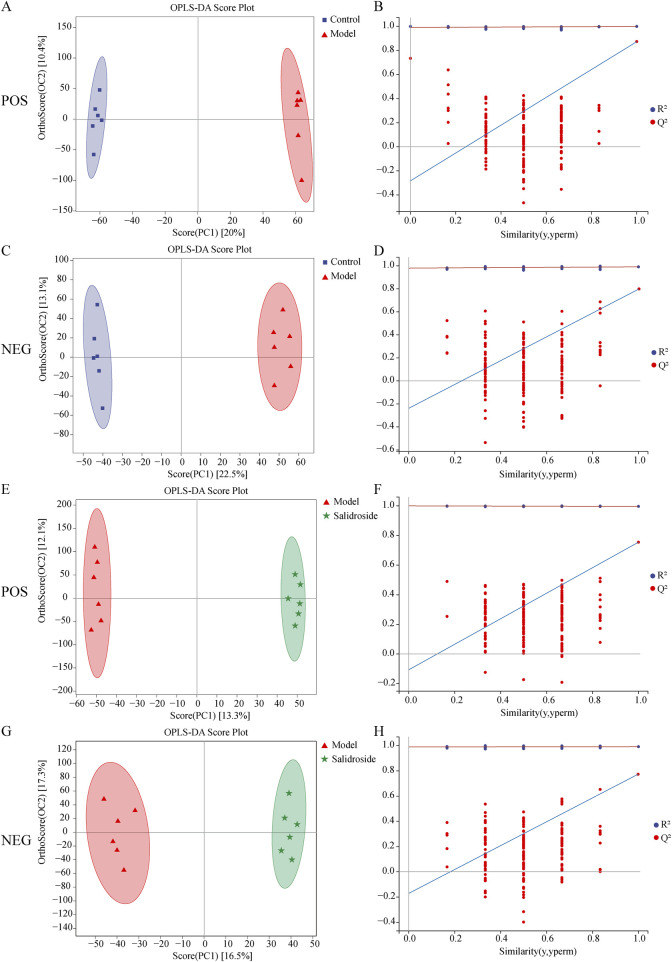
Results of OPLS-DA model in positive mode **(A)** and negative mode **(C)** using control and model group data and results of OPLS-DA model in positive mode **(E)** and negative mode **(G)** using model group and salidroside group data. Permutations Plot analysis **(B, D, F, H)**. The Q^2^ regression line’s intersection with the Y-axis was less than zero, showing that the model was valid.

As can be seen from Figure 3, the metabolic components of the three groups showed a partial separation trend, especially in the blank group and the model group, which showed that H_2_O_2_-induced TM3 cells caused differences in cell metabolites. In order to further screen the differences between groups, OPLS-DA was used to further analyze the cell metabolism samples. OPLS-DA score shows that the control group and the model group are obviously separated (positive ion mode R^2^X = 0.303, R^2^Y = 0.999, Q^2^ = 0.873; The negative ion mode R^2^X = 0.343,R^2^Y = 0.991, Q^2^ = 0.791). After salidroside treated, the model group and salidroside group were obviously separated, indicating that the metabolites in TM3 cells changed significantly after salidroside intervention (positive ion mode R^2^X = 0.229, R^2^Y = 0.997, Q^2^ = 0.746; The negative ion mode R^2^X = 0.309, R^2^Y = 0.992, Q^2^ = 0.785), as shown in Figure 4. Moreover, the regression lines of Q2 in both positive and negative ion modes intersect the negative semi-axis of *Y*-axis, which shows that the model has good stability, no over-fitting, and good prediction ability and reliability.

#### 3.3.3 Differential metabolite analysis

**FIGURE 5:**
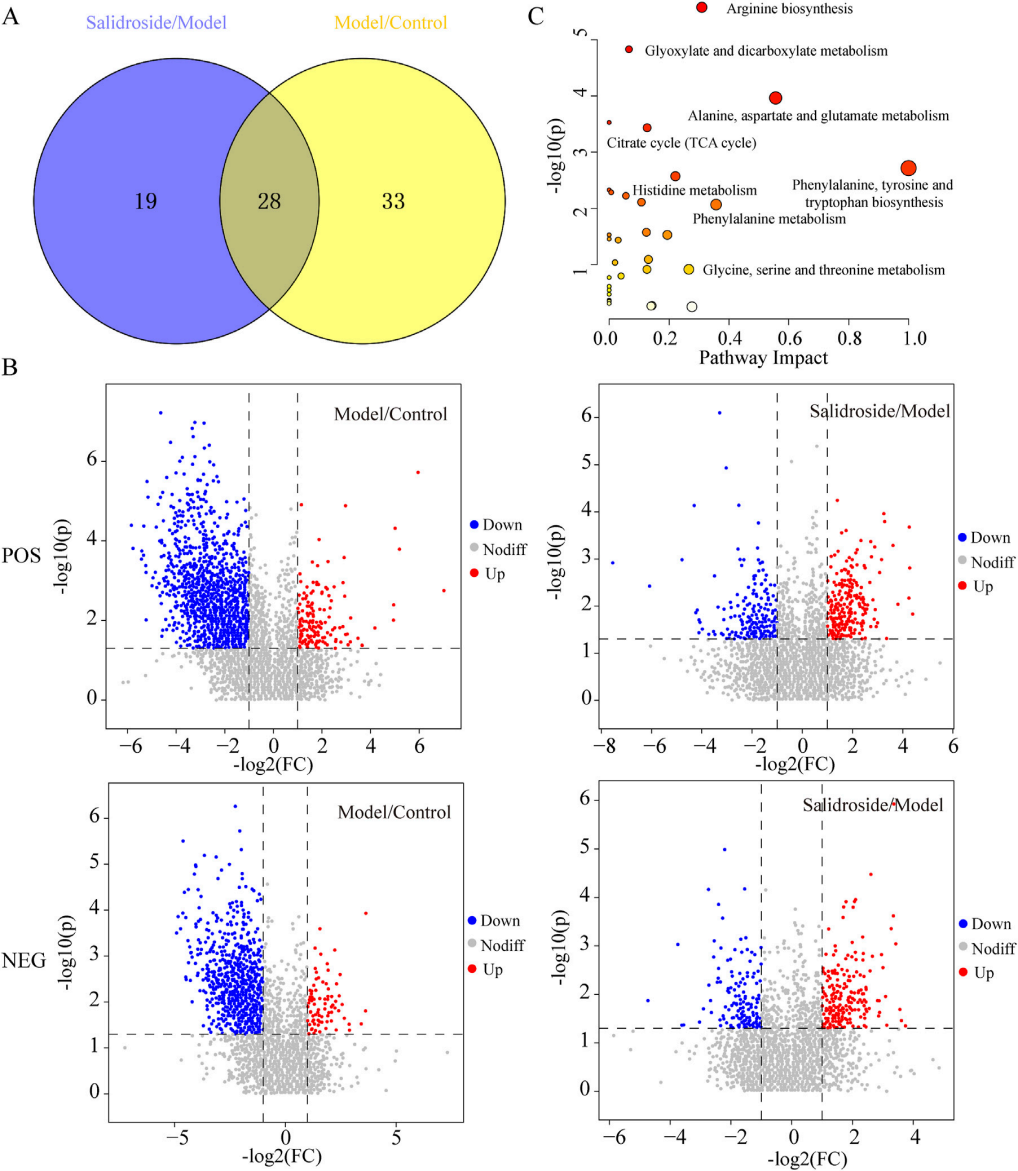
Differential metabolite analysis. **(A)** Venn diagram of differential metabolite. **(B)** Volcanic maps of Model/Control and Salidroside/Model in positive and negative ion mode. **(C)** Enrichment analysis of differential metabolite pathways.

According to OPLS-DA model, with VIP>1 and *p* < 0.05 as the standard, the different metabolites between groups were screened. The potential biomarkers were compared by Mtelin, HMDB and other metabolite databases, and 28 different metabolites were identified in positive and negative ion modes. See Figure 5A; Table 2.

**TABLE 2:** Differential metabolite analysis.

NO.	Rt (min)	m/z	Metabolites	Ion	Formula	HMDB	Model/Control	Salidroside/Model
1	0.53	147.1122	L-Lysine	[M + H] ^+^	C_6_H_14_N_2_O_2_	HMDB0000182	↓^**^	↑^**^
2	0.65	132.0761	creatine	[M + H] ^+^	C_4_H_9_N_3_O_2_	HMDB0000064	↓^*^	↑^*^
3	0.74	123.0557	Niacinamide	[M + H] ^+^	C_6_H_6_N_2_O	HMDB0001406	↓^*^	↑^**^
4	0.86	182.0807	L-tyrosine	[M + H] ^+^	C_9_H_11_NO_3_	HMDB0000158	↓^*^	↑^**^
5	0.95	243.0615	Uridine	[M-H] ^-^	C_9_H_12_N_2_O_6_	HMDB0000296	↓^*^	↑^*^
11	0.97	268.1029	Adenosine	[M + H] ^+^	C_10_H_13_N_5_O_4_	HMDB0000050	↓^*^	↑^**^
6	1.03	608.0886	UDP-N-acetylglucosamine	[M + H] ^+^	C_17_H_27_N_3_O_17_P_2_	HMDB0000290	↓^**^	↑^*^
7	1.25	106.0499	Serine	[M + H] ^+^	C_3_H_7_NO_3_	HMDB0003406	↓^*^	↑^*^
8	1.45	269.0874	Inosine	[M + H] ^+^	C_10_H_12_N_4_O_5_	HMDB0000195	↓^*^	↑^*^
9	1.68	150.0588	L-Methionine	[M + H] ^+^	C_5_H_11_NO_2_S	HMDB0000696	↓^**^	↑^**^
10	2.87	229.1539	Leucylproline	[M + H] ^+^	C_11_H_20_N_2_O_3_	HMDB0011175	↓^*^	↑^*^
12	3.42	130.086	Leucine	[M−H]−	C_6_H_13_NO_2_	HMDB0000687	↓^*^	↑^*^
13	3.58	175.1182	L-Arginine	[M + H] ^+^	C_6_H_14_N_4_O_2_	HMDB0000517	↓^**^	↑^*^
14	3.83	166.0867	L-Phenylalanine	[M + H] ^+^	C_9_H_11_NO_2_	HMDB0000159	↑^*^	↓^*^
15	5.76	220.1183	Pantothenic acid	[M + H] ^+^	C_9_H_17_NO_5_	HMDB0000210	↑^*^	↓^*^
16	5.94	148.0607	L-Glutamic acid	[M + H] ^+^	C_5_H_9_NO_4_	HMDB0000148	↓^*^	↑^*^
17	6.53	247.1279	Glutamylvaline	[M + H] ^+^	C_10_H_18_N_2_O_5_	HMDB0028832	↓^*^	↑^*^
18	6.59	291.1302	Argininosuccinic acid	[M + H] ^+^	C_10_H_18_N_4_O_6_	HMDB0000052	↓^*^	↑^**^
19	8.76	133.0135	L-Malic acid	[M − H] ^−^	C_4_H_6_O_5_	HMDB0000156	↓^*^	↑^**^
20	9.58	119.0337	Succinic acid	[M + H] ^+^	C_4_H_6_O_4_	HMDB0000254	↓^*^	↑^*^
21	9.71	137.0452	Hypoxanthine	[M + H] ^+^	C_5_H_4_N_4_O	HMDB0000157	↓^**^	↑^*^
22	10.32	203.2233	Spermine	[M + H] ^+^	C_10_H_26_N_4_	HMDB0001256	↓^*^	↑^*^
23	14.72	134.0445	L-Aspartic acid	[M + H] ^+^	C_4_H_7_NO_4_	HMDB0000191	↓^*^	↑^**^
24	15.19	145.0611	L-Glutamine	[M−H] ^−^	C_5_H_10_N_2_O_3_	HMDB0000641	↓^*^	↑^*^
25	16.67	151.0253	Xanthine	[M−H] ^−^	C_5_H_4_N_4_O_2_	HMDB0000292	↑^*^	↓^*^
26	17.53	166.9742	Phosphoenolpyruvic acid	[M−H] ^−^	C_3_H_5_O_6_P	HMDB0000263	↓^*^	↑^**^
27	18.41	175.0241	cis-Aconitic acid	[M + H] ^+^	C_6_H_6_O_6_	HMDB0000072	↓^*^	↑^*^
28	21.24	303.2315	Arachidonic acid	[M−H] ^−^	C_20_H_32_O_2_	HMDB0001043	↓^**^	↑^*^

#### 3.3.4 Metabolic pathway analysis of differential metabolites

**FIGURE 6:**
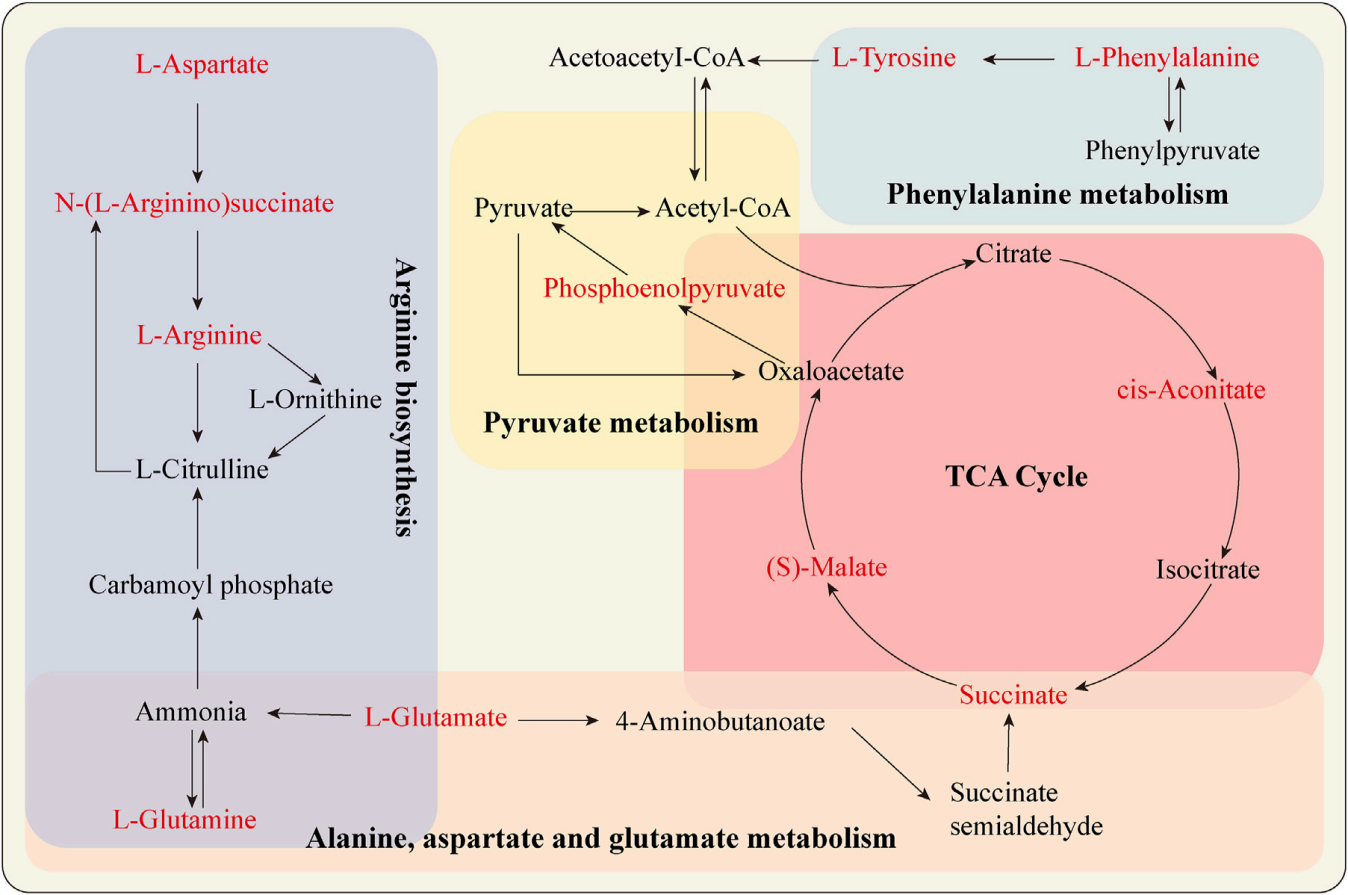
Analysis of metabolic pathways of differential metabolites.

By using MetaboAnalyst 5.0, the metabolic pathway topology analysis and enrichment analysis of the differential metabolites were carried out, and the following metabolic pathways were selected as the key related metabolic pathways, as shown in Figure 5C. The results showed that salidroside mainly affected metabolic pathways such as Arginine biosynthesis, Alanine, aspartate and glutamate metabolism, Citrate cycle (TCA cycle), Phenylalanine metabolism and Pyruvate metabolism. Furthermore, the metabolic pathway of salidroside affecting testosterone secretion in TM3 cells was constructed through metabolic pathway (As shown in Figure 6).

### 3.4 Network pharmacological analysis

#### 3.4.1 Analysis of metabolic targets of differential metabolites

**FIGURE 7:**
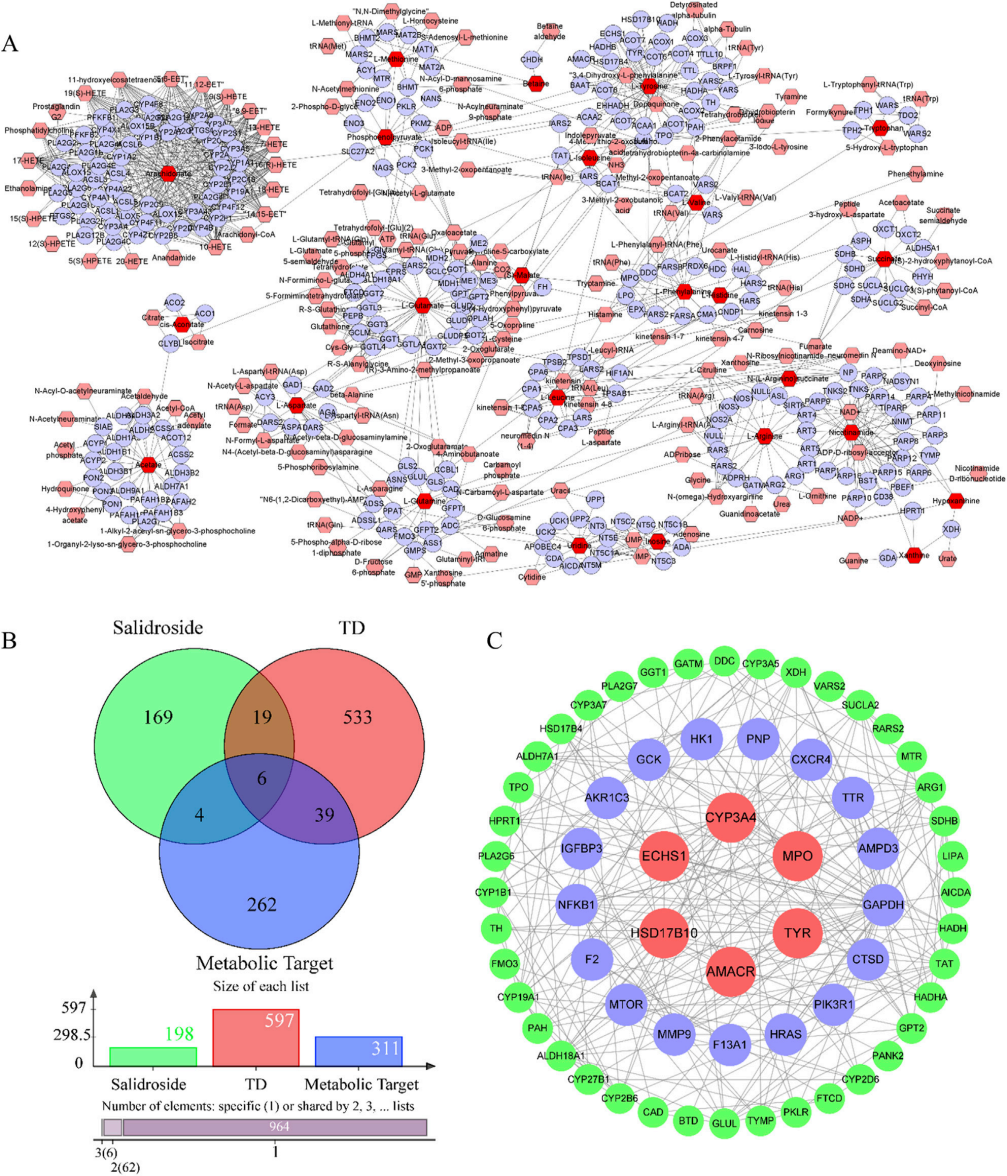
Analysis of network pharmacology. **(A)** Metabolic targets of differential metabolites. **(B)** Intersecting targets. **(C)** PPI network.

The metabolic targets of different metabolites were analyzed based on Cytoscape 3.8.0 software, and 312 metabolic targets were obtained, as shown in Figure 7A.

#### 3.4.2 Acquisition of metabolic target, salidroside and common targets of disease

Salidroside and testosterone deficiency predicted 199 and 597 related targets respectively. A total of six common targets were obtained by cross-mapping with metabolic targets as potential targets for further research. As shown in Figures 7B, C.

#### 3.4.3 GO and KEGG analysis

**FIGURE 8:**
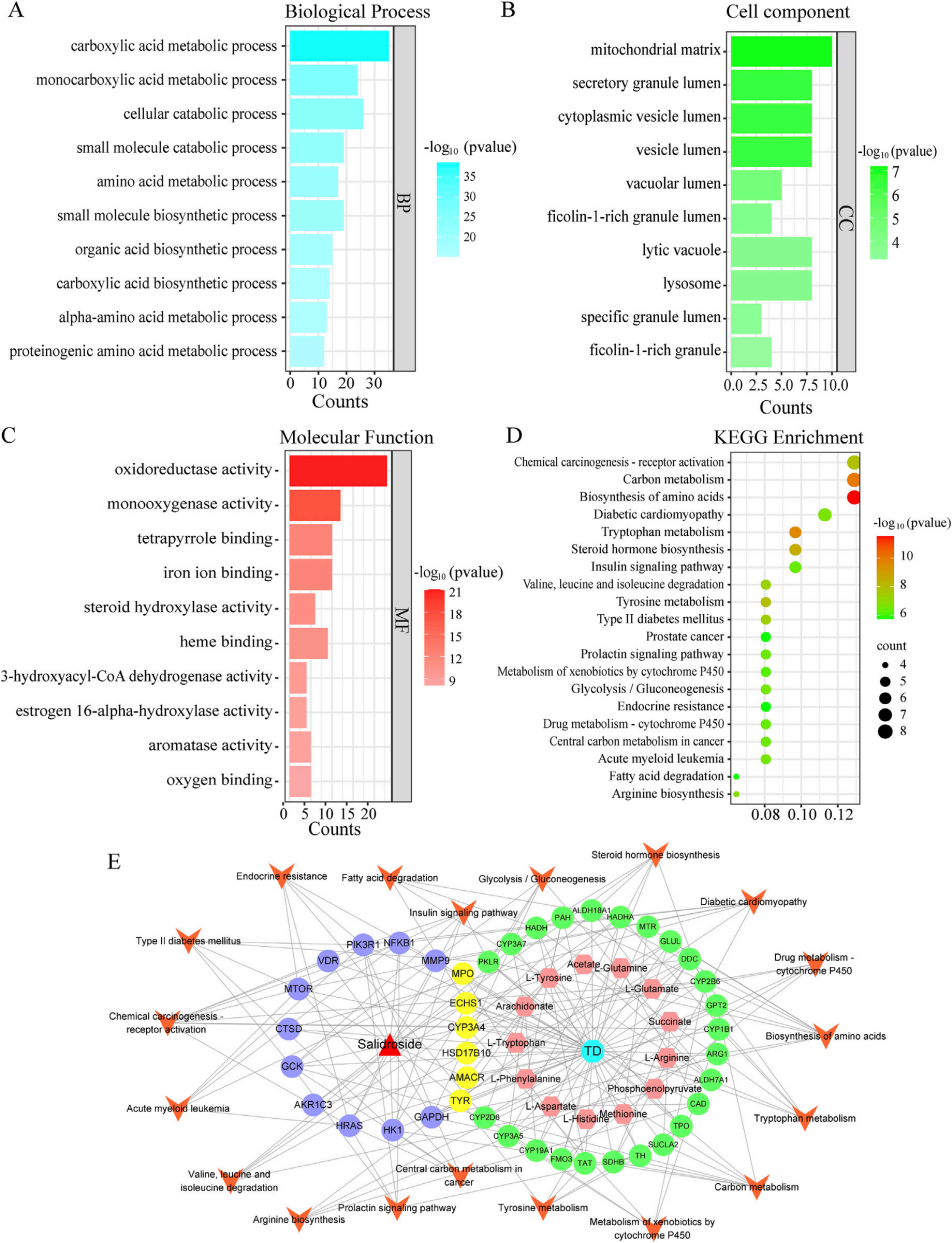
GO and KEGG analysis. **(A)** Biological process. **(B)** Cell component. **(C)** Molecular function. **(D)** Kegg enrichment. **(E)** “drug-metabolite-target-pathway-disease” network.

In order to further clarify the biological process related to the core target, GO and KEGG enrichment analysis were carried out (As shown in Figures 8A–D). GO analysis mainly involves carboxylic acid metabolism, amino acid metabolism, oxidoreductase activity and steroid hydroxylase activity. KEGG enrichment analysis mainly involves amino acid biosynthesis, steroid hormone biosynthesis, glycolysis/gluconeogenesis, arginine biosynthesis and other signal pathways. Construct “component-target-pathway-disease” network, as shown in Figure 8E.

### 3.5 Molecular docking

**FIGURE 9:**
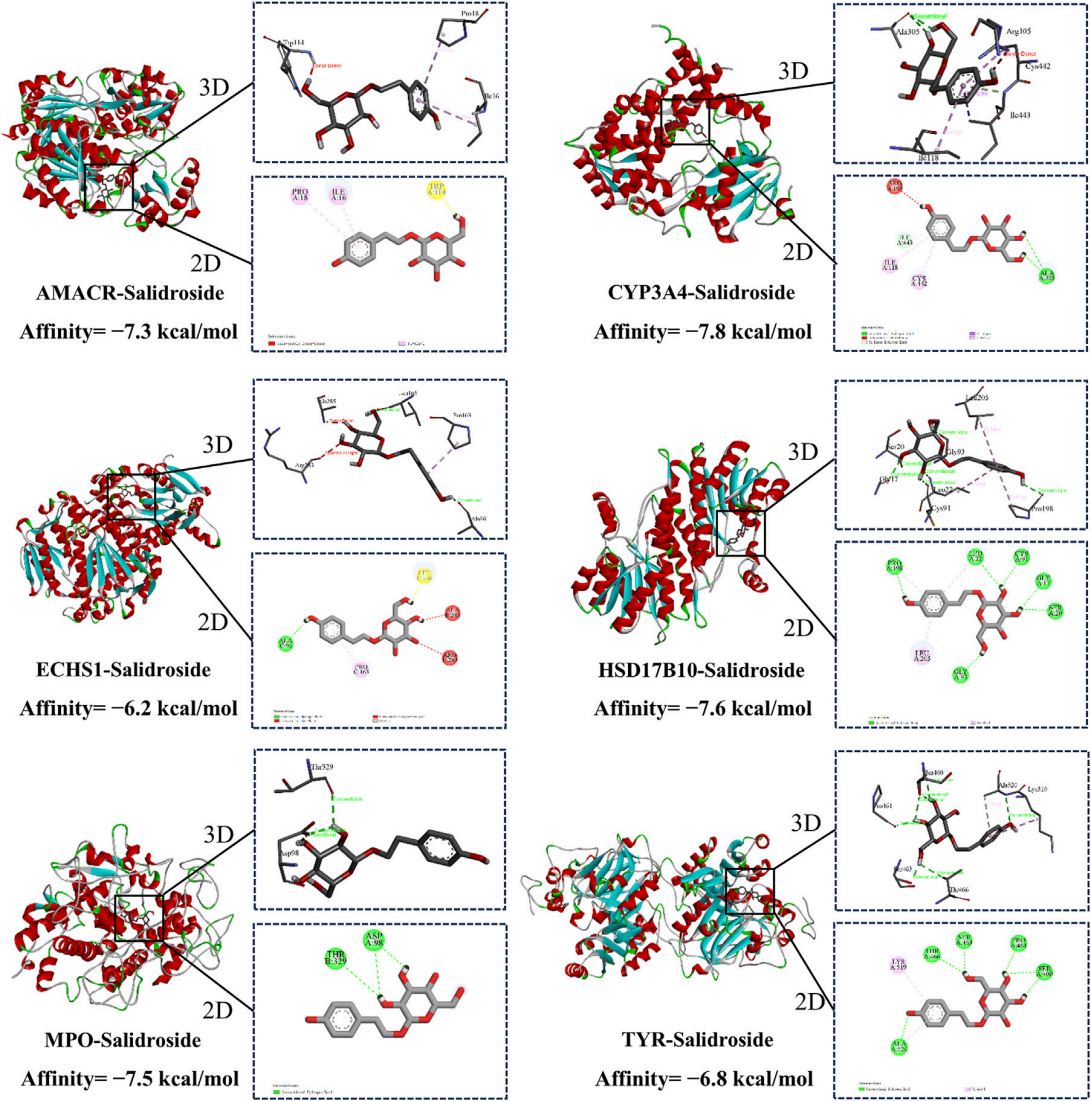
Molecular docking.

Molecular docking was used to evaluate the affinity between salidroside and the core target. It is generally believed that the lower the binding energy, the better the docking effect, indicating that the more stable the binding between active component ligand molecules and core target receptor proteins, the higher the affinity. The binding energies of the compounds were calculated by AutoDock Vina software. The results showed that the binding energies were lower than −5 kcal/mol and the compounds had good affinity. Finally, the visual analysis was performed with the Discovery Studio 2019 software, as shown in Figure 9.

### 3.6 Molecular dynamics simulation

**FIGURE 10:**
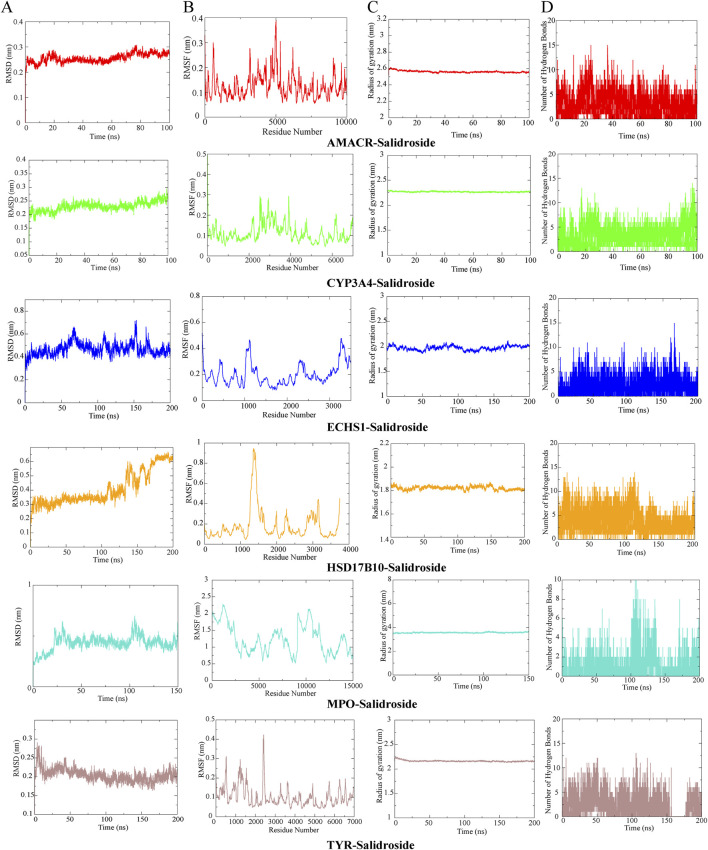
Molecular dynamics simulation. **(A)** RMSD analysis. **(B)** RMSF analysis. **(C)** RG analysis. **(D)** Hydrogen bond analysis.

Through metabolomics combined with network pharmacological analysis and molecular docking results, the core targets AMACR, CYP3A4, ECHS1, HSD17B10, MPO, TYR and salidroside were selected for molecular dynamics simulation verification. RMSD value is used to evaluate whether the simulation system reaches a stable state, and the RMSD value within 1 nm indicates the relative stability of protein-ligand interaction in physiological environment, and the results are shown in Figure 10A. To analyze the Molecular dynamics of the residues of various proteins in the complex during the simulation process, the RMSF values of all protein residues during the simulation process were calculated, the greater the interaction between the residues of the protein and the small molecule, see Figure 10B. RG represents the compactness of protein structure in the simulation process, as shown in Figure 10C, and salidroside is closely bound to each target. Hydrogen bonding is one of the strongest noncovalent interactions in the binding process between small molecular compounds and proteins. The number of hydrogen bonds reflects the strength of protein-ligand binding. As shown in Figure 10D, hydrogen bonding plays an important role in the stable existence of six complexes. Results It was verified that all targets and corresponding compounds could bind stably.

### 3.7 RT-qPCR analysis

**FIGURE 11:**
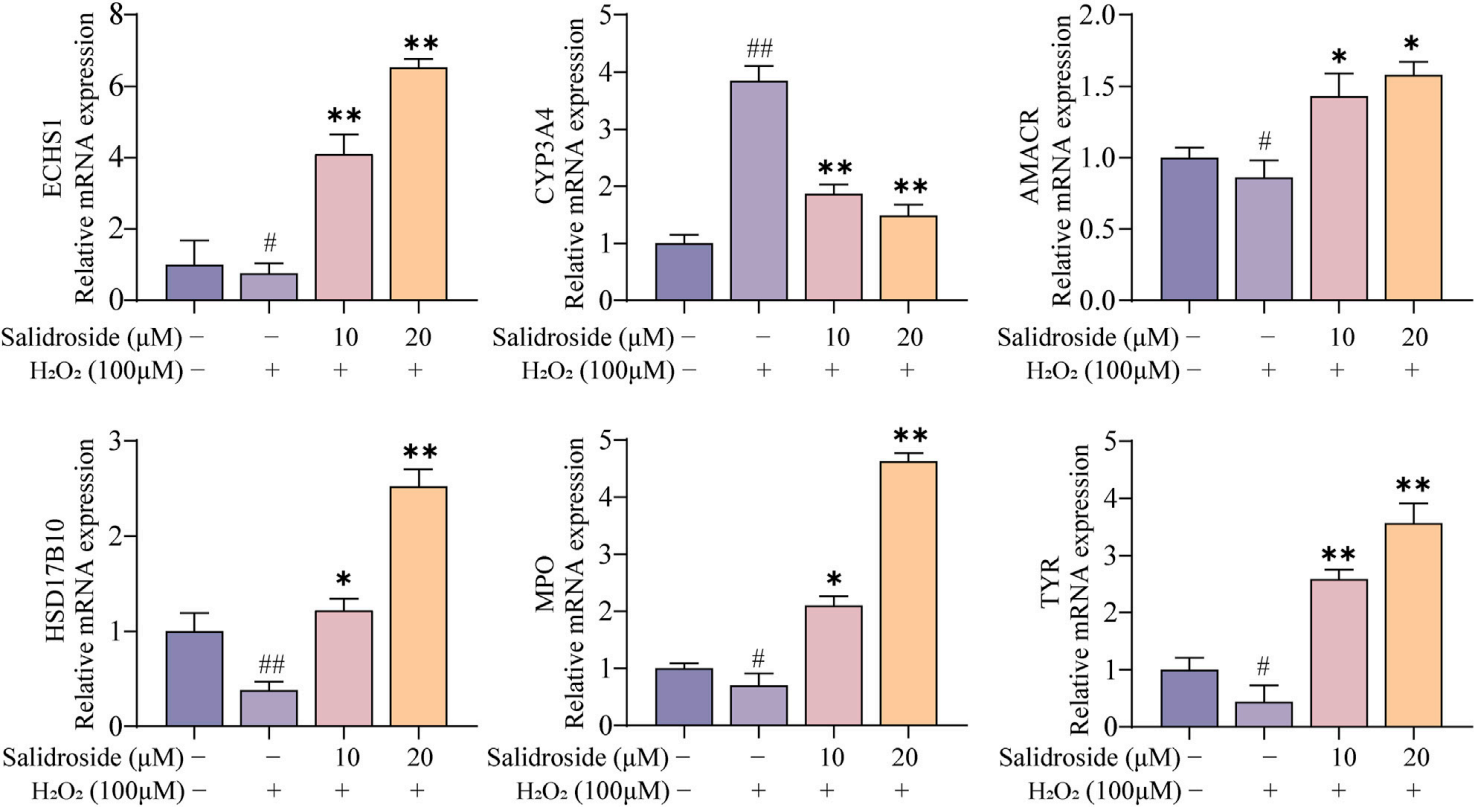
RT-qPCR analysis. (mean ± SD, n = 3). Compared with the control group, ^#^p < 0.05, ^##^p < 0.01; Compared with the model group, *p < 0.05, **p < 0.01.

As shown in Figure 11. RT-qPCR results showed that the core targets were AMACR, CYP3A4, ECHS1, HSD17B10, MPO and TYR. Compared with the control group, the levels of AMACR, ECHS1, HSD17B10, MPO and TYR in the model group decreased significantly (*p* < 0.05, *p* < 0.01), while the levels of CYP3A4 increased (*p* < 0.01). Compared with the model group, the expressions of AMACR, ECHS1, HSD17B10, MPO and TYR in salidroside treated group increased significantly (*p* < 0.05, *p* < 0.01), while the expression of CYP3A4 decreased (*p* < 0.05, *p* < 0.01). It further shows that salidroside can promote testosterone secretion through the above six core targets.

## 4 Discussion

The testicles are critical reproductive organs in male animals, primarily responsible for the secretion of androgens, with testosterone being a principal hormone. Testosterone synthesis involves a series of enzymatic reactions and is predominantly secreted by the Leydig cells within the testes (Nassar and Leslie, 2023). A deficiency in testosterone can lead to conditions such as sexual dysfunction and metabolic disorders. Conversely, enhanced testosterone secretion has been shown to ameliorate hypogonadism (Groti Antonič and Zitzmann, 2024), improve glycemic control in diabetes (Ashour, 2024), treat atherosclerosis in middle-aged and elderly individuals (Albuquerque et al., 2020), and address osteoporosis (Mbiydzenyuy and Qulu, 2024). Research has indicated that oxidative stress-induced damage to Leydig cells in the testes is a significant contributor to male infertility. In this study, we developed an oxidative damage model of TM3 cells using H_2_O_2_ and discovered that salidroside enhances TM3 cell activity. This enhancement is evidenced by a significant increase in testosterone, SOD, and GSH levels, alongside a reduction in ROS and MDA levels. It shows that salidroside can effectively improve oxidative stress, reduce oxidative damage, protect TM3 cells and promote testosterone secretion.

It is worth noting, but there is no exact mechanism that salidroside promotes testosterone secretion by improving oxidative stress. Metabolomics serves as a tool to investigate the internal alterations within organisms, with metabolites representing the ultimate products of gene transcription and the foundational material basis of organismal phenotypes (Wishart, 2019). In this study, cell metabolomics was employed to delineate the metabolic pathway modulated by salidroside, as inferred from the downstream small molecular differential metabolites. Salidroside has been identified to influence the production of 28 distinct metabolites, such as arginine, aspartic acid, leucine, and arachidonic acid, among others. These metabolites are primarily associated with metabolic pathways including arginine biosynthesis, alanine, aspartate, and glutamate metabolism, the citrate cycle (TCA cycle), phenylalanine metabolism, and pyruvate metabolism. These findings demonstrate that salidroside plays a role in multiple metabolic pathways, contributing to the regulation of testosterone secretion.

Interestingly, arginine biosynthesis is very important for epididymal sperm maturation (Tsuchiya et al., 2023; Wu et al., 2024). L-arginine, a precursor of nitric oxide, is essential for maintaining normal cellular nitric oxide levels (Wu et al., 2021). Nitric oxide is implicated in the regulation of mitochondrial biogenesis, the respiratory chain, and oxidative stress. In the testes, D-aspartic acid has been shown to enhance testosterone release, upregulate androgen receptor expression, and downregulate estrogen receptor expression (Roshanzamir and Safavi, 2017; Falvo et al., 2024). Furthermore, tyrosine and phenylalanine serve as precursors in the biosynthesis of neurotransmitters and hormones (Schenck and Maeda, 2018; Lu et al., 2024). Phenylalanine, classified as an essential aromatic amino acid, is metabolized by phenylalanine hydroxylase in bodily tissues, including the liver, ultimately leading to the production of dopamine (Wang et al., 2021). Dopamine plays a regulatory role in the hypothalamic-pituitary-gonadal axis, thereby supplying the requisite substrates for testosterone synthesis (Salonia et al., 2019). In addition, pyruvate is the product of glycolytic pathway, which is located at the junction of anaerobic glycolysis and aerobic oxidation metabolism, and plays an important role in maintaining the metabolism of tissues and cells and ensuring energy (Liang et al., 2021).

To further investigate the mechanisms by which salidroside enhances testosterone secretion, this study employed an integrative approach combining metabolomics and network pharmacology. The research examined the correlation between upstream targets and downstream differential metabolites, culminating in the construction of a “drug-metabolite-target-pathway-disease” network. The findings indicated that the promotion of testosterone secretion by salidroside is closely associated with six key targets: AMACR, CYP3A4, ECHS1, HSD17B10, MPO, and TYR. It is noteworthy that CYP3A4, a cytochrome P450 enzyme, plays a crucial role in the metabolism of testosterone within the body, facilitating its conversion into metabolites such as 6β-hydroxytestosterone, which subsequently diminishes the biological activity of testosterone (Christiansen et al., 2011). Additionally, HSD17B10 (hydroxysteroid 17-β dehydrogenase 10) is a mitochondrial enzyme implicated in the metabolic processing of steroid hormones (He et al., 2023). During testosterone biosynthesis, HSD17B10 primarily catalyzes the conversion of androstenedione to testosterone. MPO is an enzyme present in neutrophils and eosinophils that plays a crucial role in mitigating oxidative damage and preserving cellular function (Michaeloudes et al., 2022). It serves to protect interstitial cells from oxidative stress and facilitates the secretion of testosterone. Furthermore, AMACR and ECHS1 are involved in the regulation of cholesterol metabolism, thereby influencing testosterone production (Hu et al., 2022; Mojanaga et al., 2024).

Subsequently, the impact of salidroside on the targets AMACR, CYP3A4, ECHS1, HSD17B10, MPO, and TYR was examined through bioinformatics analysis and cellular experiments. Molecular docking studies revealed that the binding energy was lower than −5 kcal/mol, indicating a strong affinity, and its stability was confirmed via molecular dynamics simulations. Furthermore, RT-qPCR results demonstrated that salidroside significantly upregulated the expression levels of AMACR, ECHS1, HSD17B10, MPO, and TYR, while downregulating the expression of CYP3A4. It further strengthens the important position that salidroside may act on the above-mentioned core targets in promoting testosterone secretion.

## 5 Conclusion

In summary, this study utilized the TM3 cell model, induced by salidroside and H_2_O_2_, as the primary research focus. An integrative approach combining metabolomics and network pharmacology was employed to investigate the relationship between salidroside and the biological functions of the organism at a systemic level. The study analyzed the differential metabolites, core targets, and metabolic pathways associated with salidroside’s promotion of testosterone secretion. However, it is worth noting that this study provides a preliminary theoretical and experimental basis for the study of the mechanism and clinical application of salidroside in promoting testosterone secretion, and also provides a new research idea and direction for the follow-up study of pharmacodynamic substances.

## Data Availability

The original contributions presented in the study are included in the article/supplementary material, further inquiries can be directed to the corresponding authors.
