# Supplementary figures and images for: Mechanism of salidroside promoting testosterone secretion induced by H2O2 in TM3 Leydig cells based on metabolomics and network pharmacology
Source: Front Chem. 2025 Feb 27;13:1544876. doi: 10.3389/fchem.2025.1544876 (PMC11904911; doi:10.3389/fchem.2025.1544876)

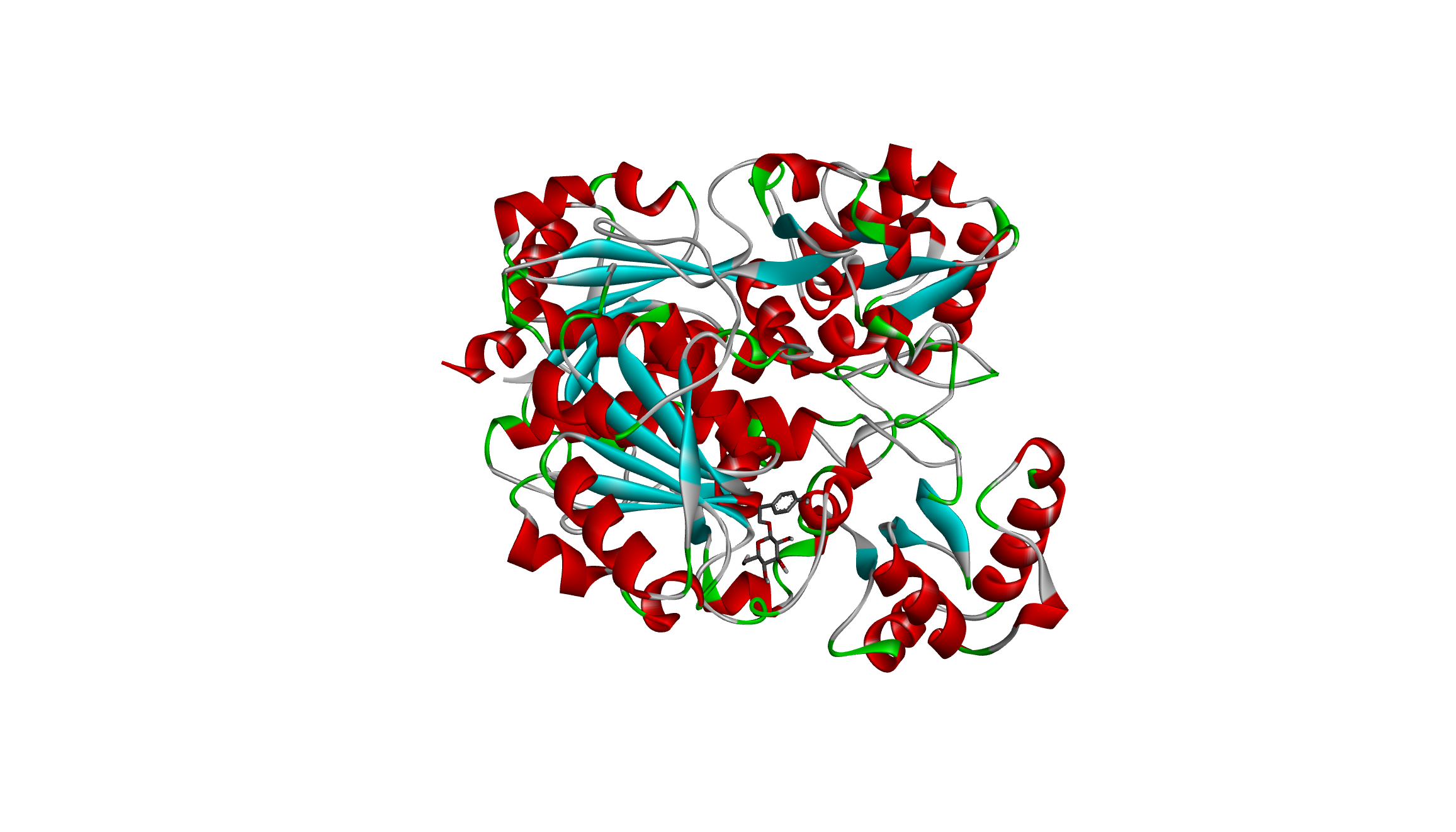

Supplement: Supplementary file 1 [file DataSheet1.ZIP › Supplementary document/Molecular docking/Figure/AMACR-1.png]

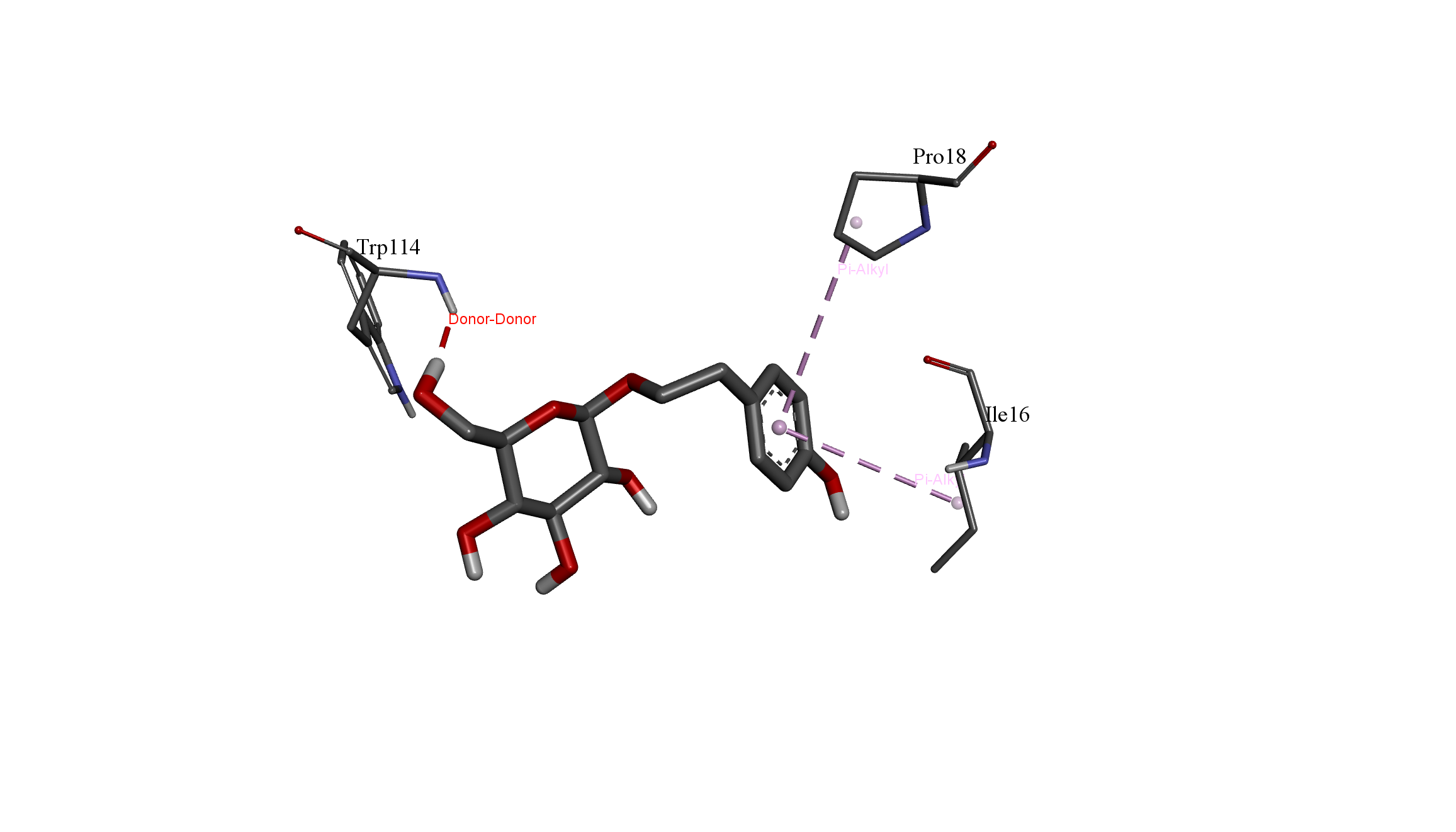

Supplement: Supplementary file 1 [file DataSheet1.ZIP › Supplementary document/Molecular docking/Figure/AMACR-2.png]

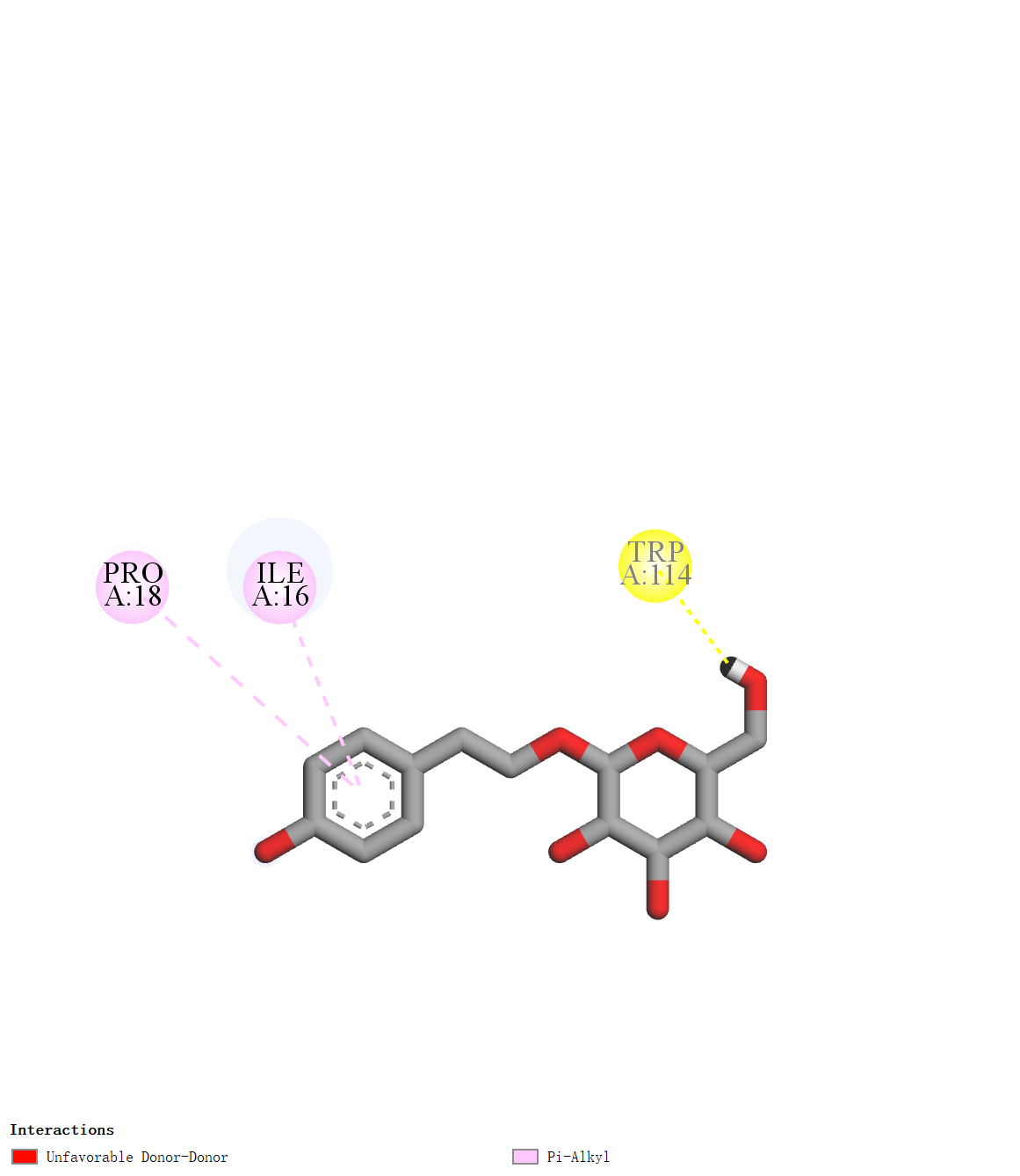

Supplement: Supplementary file 1 [file DataSheet1.ZIP › Supplementary document/Molecular docking/Figure/AMACR-3.png]

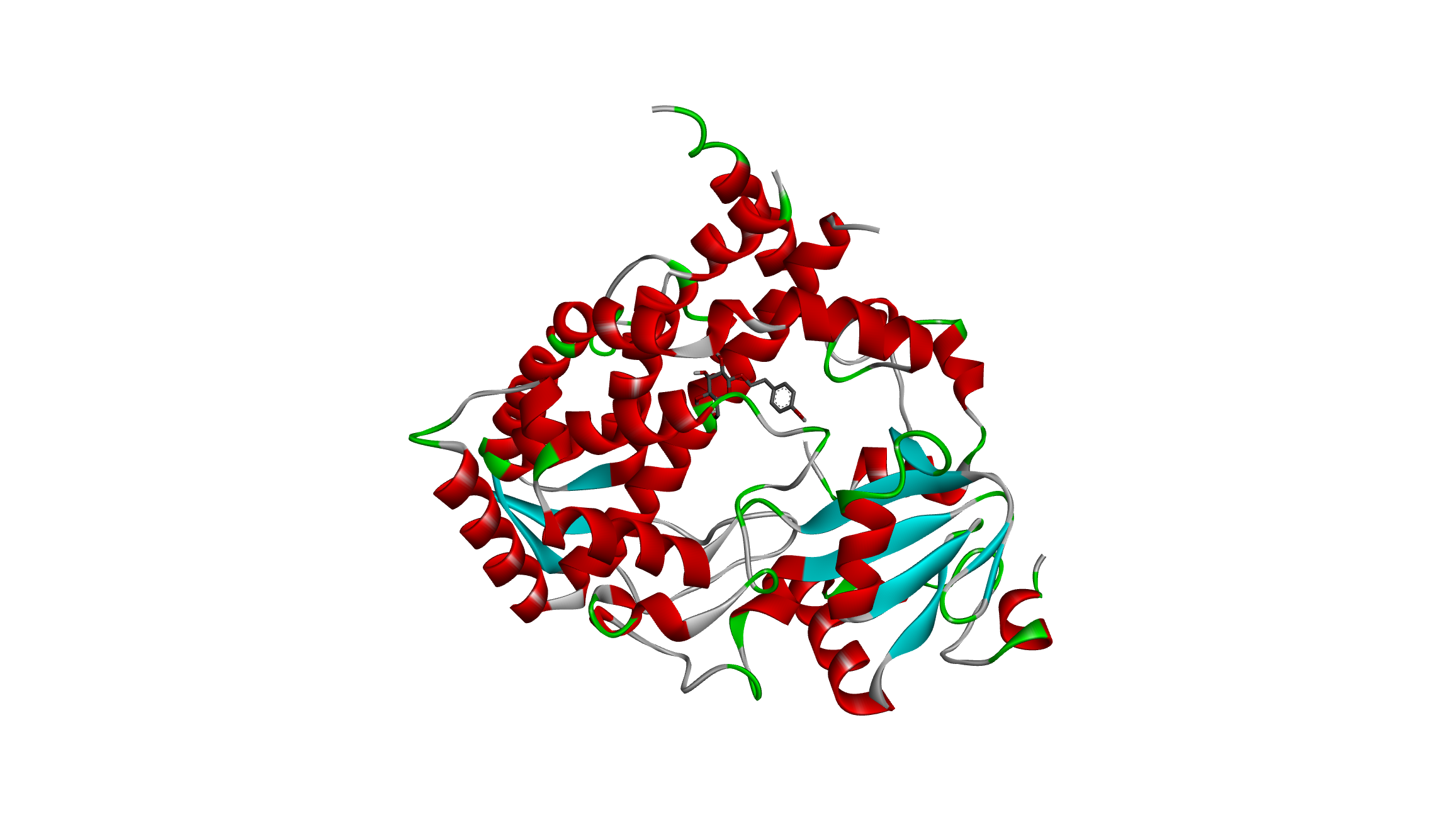

Supplement: Supplementary file 1 [file DataSheet1.ZIP › Supplementary document/Molecular docking/Figure/CYP3A4-1.png]

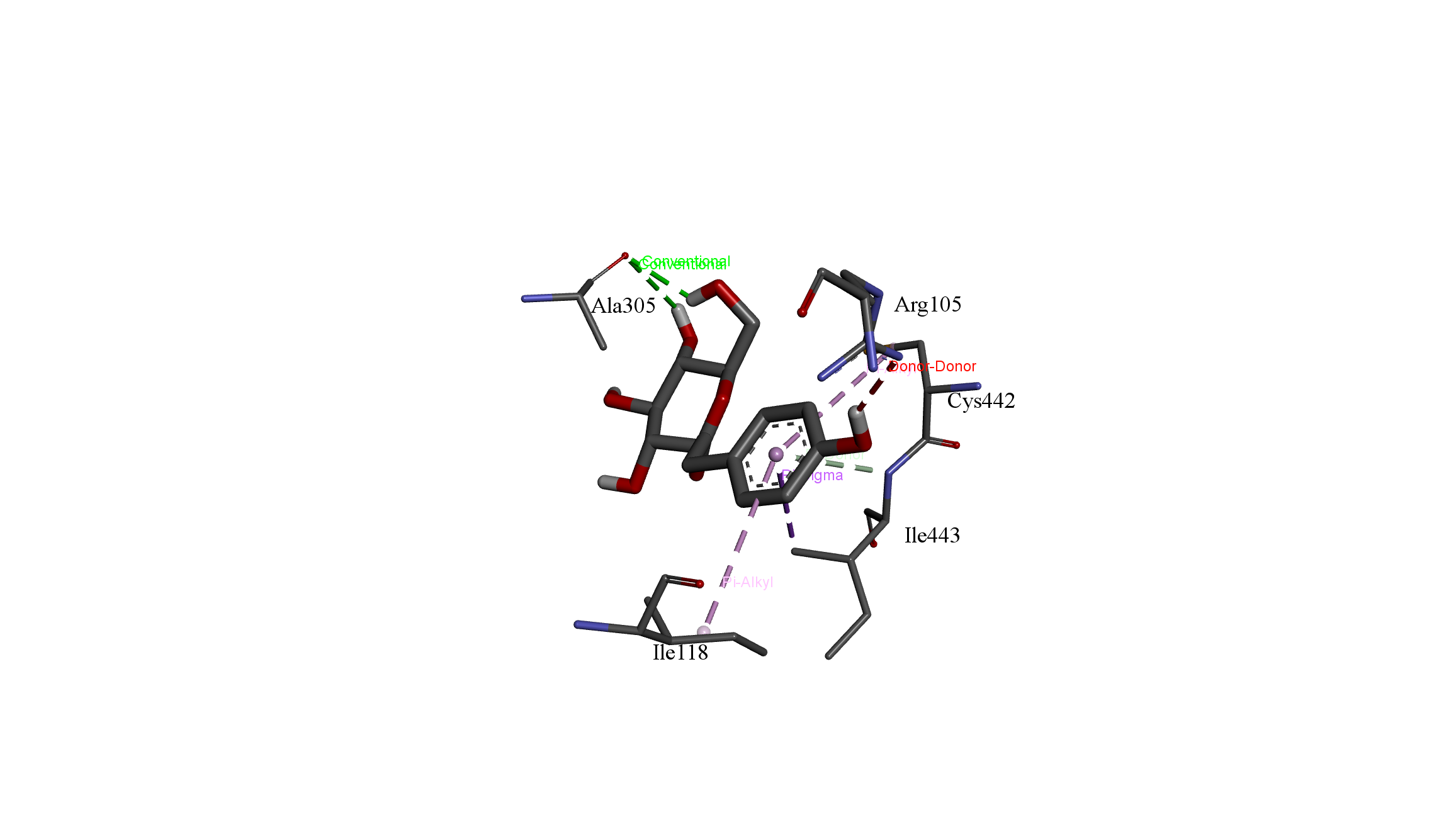

Supplement: Supplementary file 1 [file DataSheet1.ZIP › Supplementary document/Molecular docking/Figure/CYP3A4-2.png]

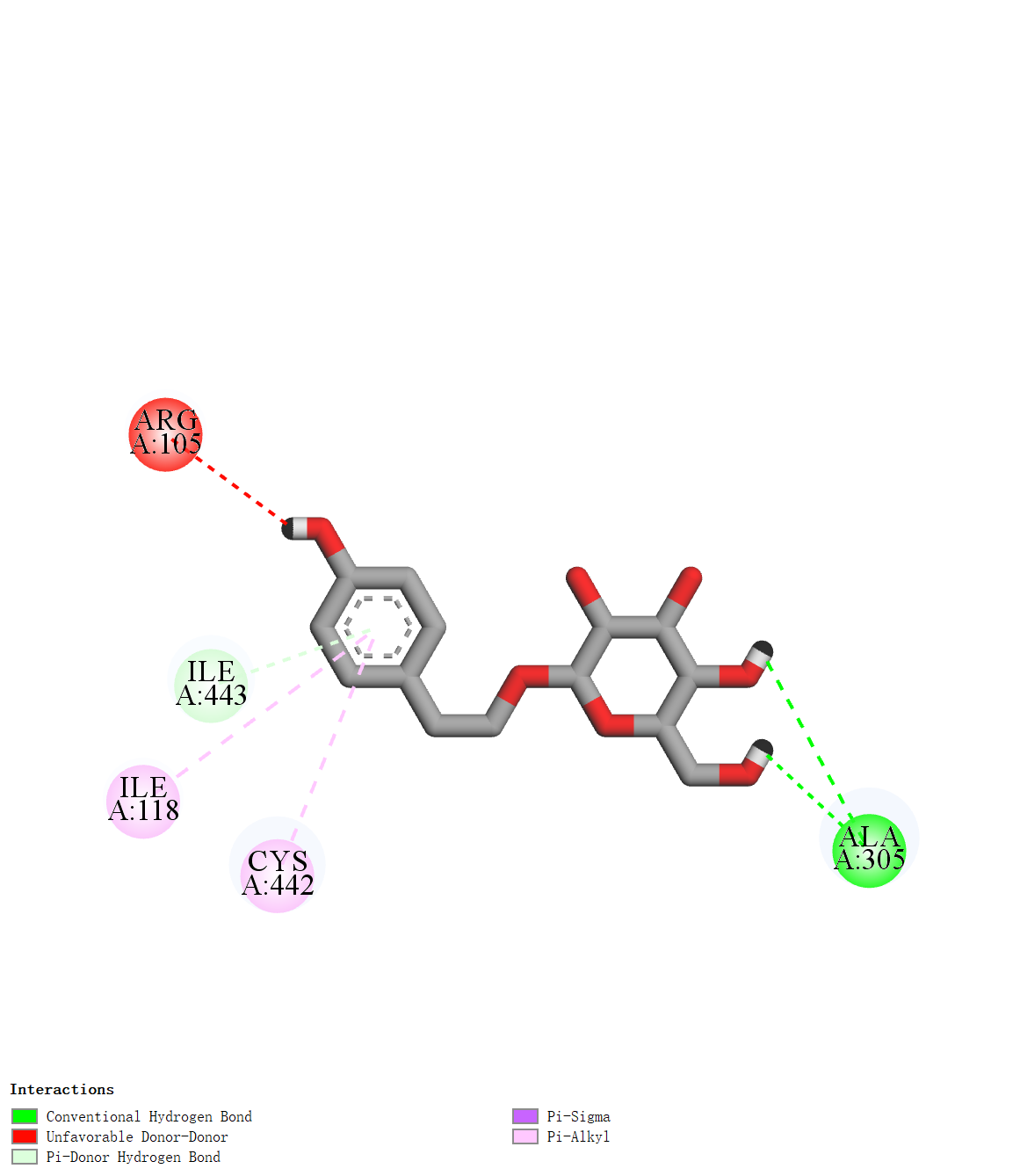

Supplement: Supplementary file 1 [file DataSheet1.ZIP › Supplementary document/Molecular docking/Figure/CYP3A4-3.png]

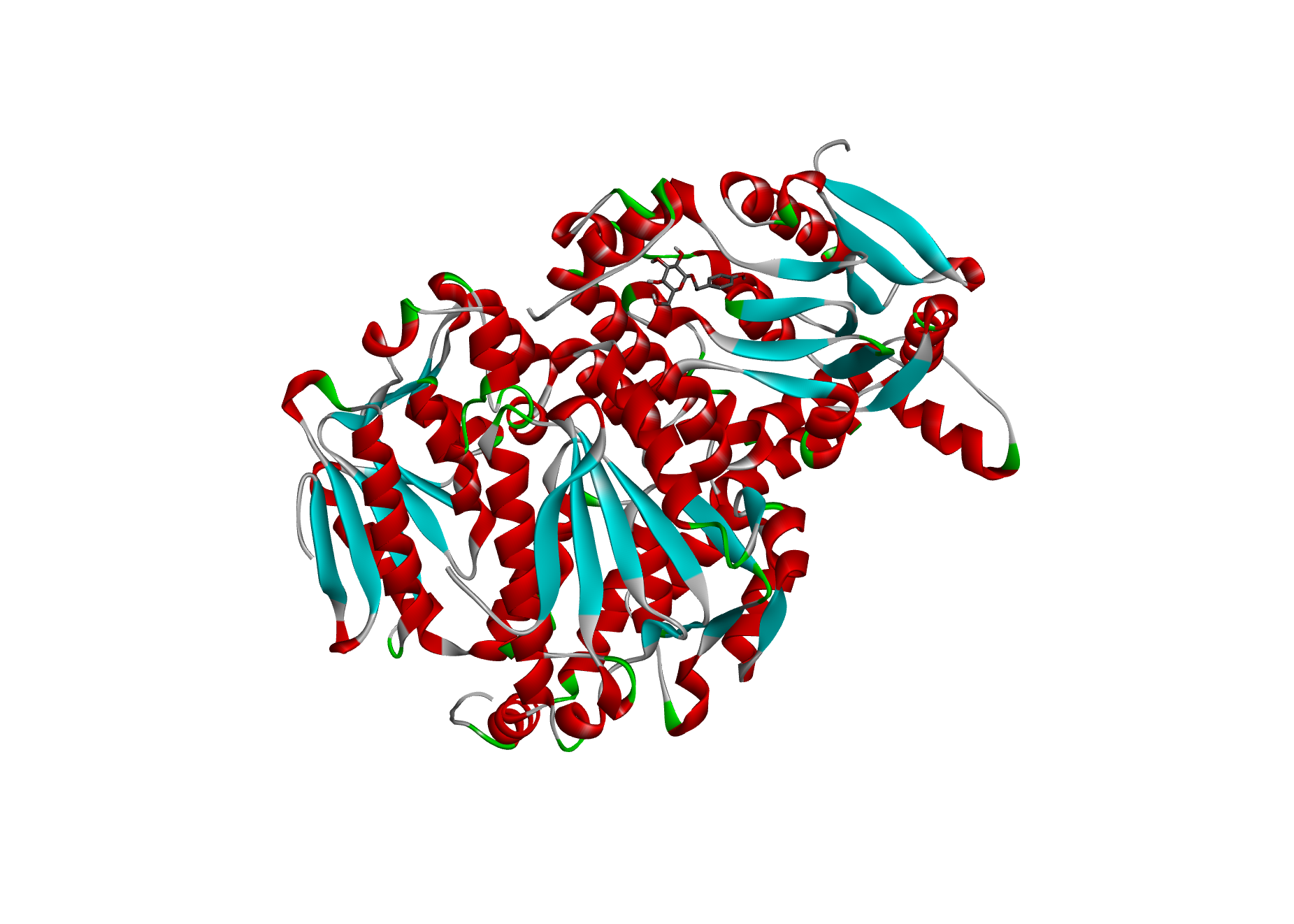

Supplement: Supplementary file 1 [file DataSheet1.ZIP › Supplementary document/Molecular docking/Figure/ECHS1-1.png]

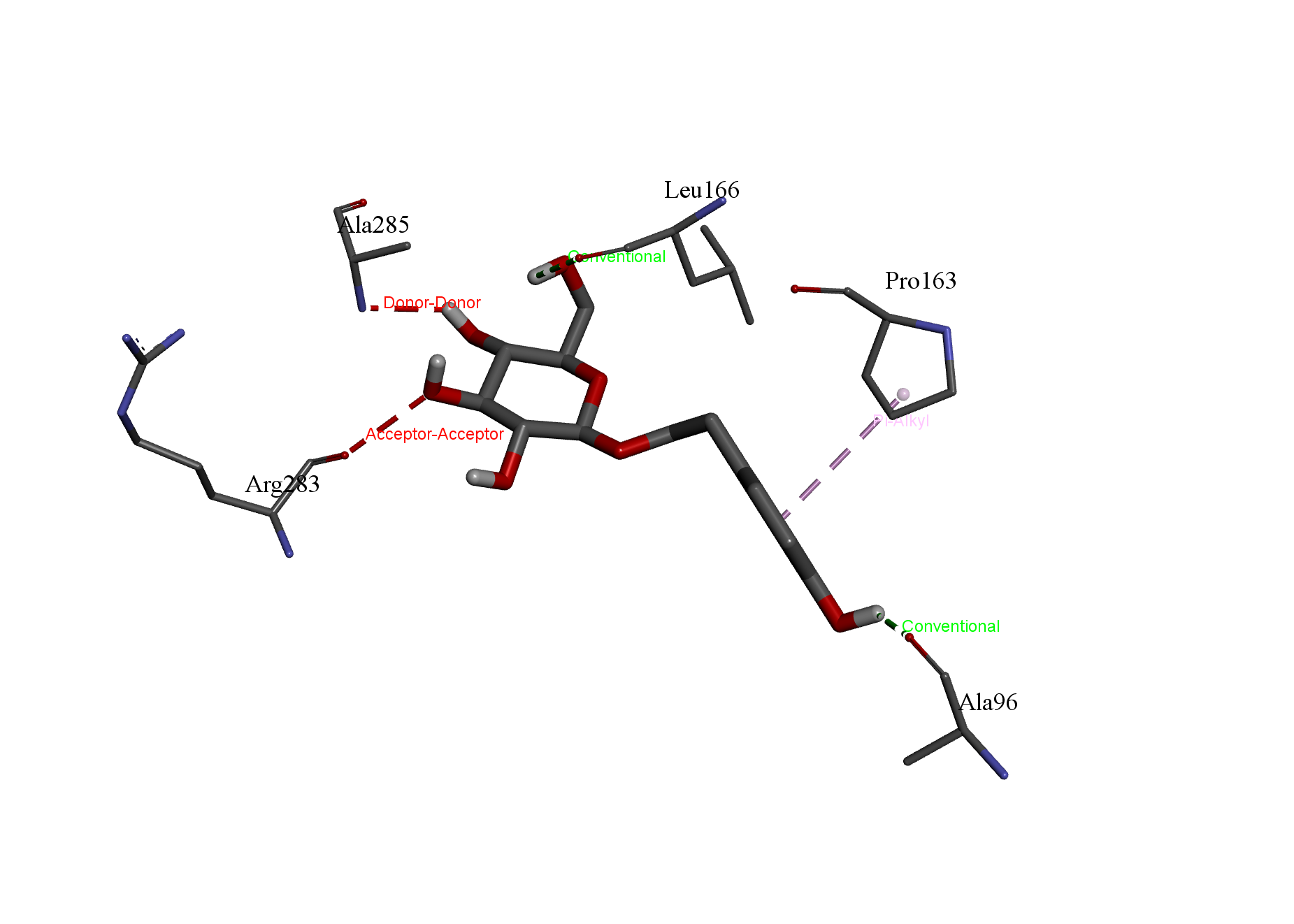

Supplement: Supplementary file 1 [file DataSheet1.ZIP › Supplementary document/Molecular docking/Figure/ECHS1-2.png]

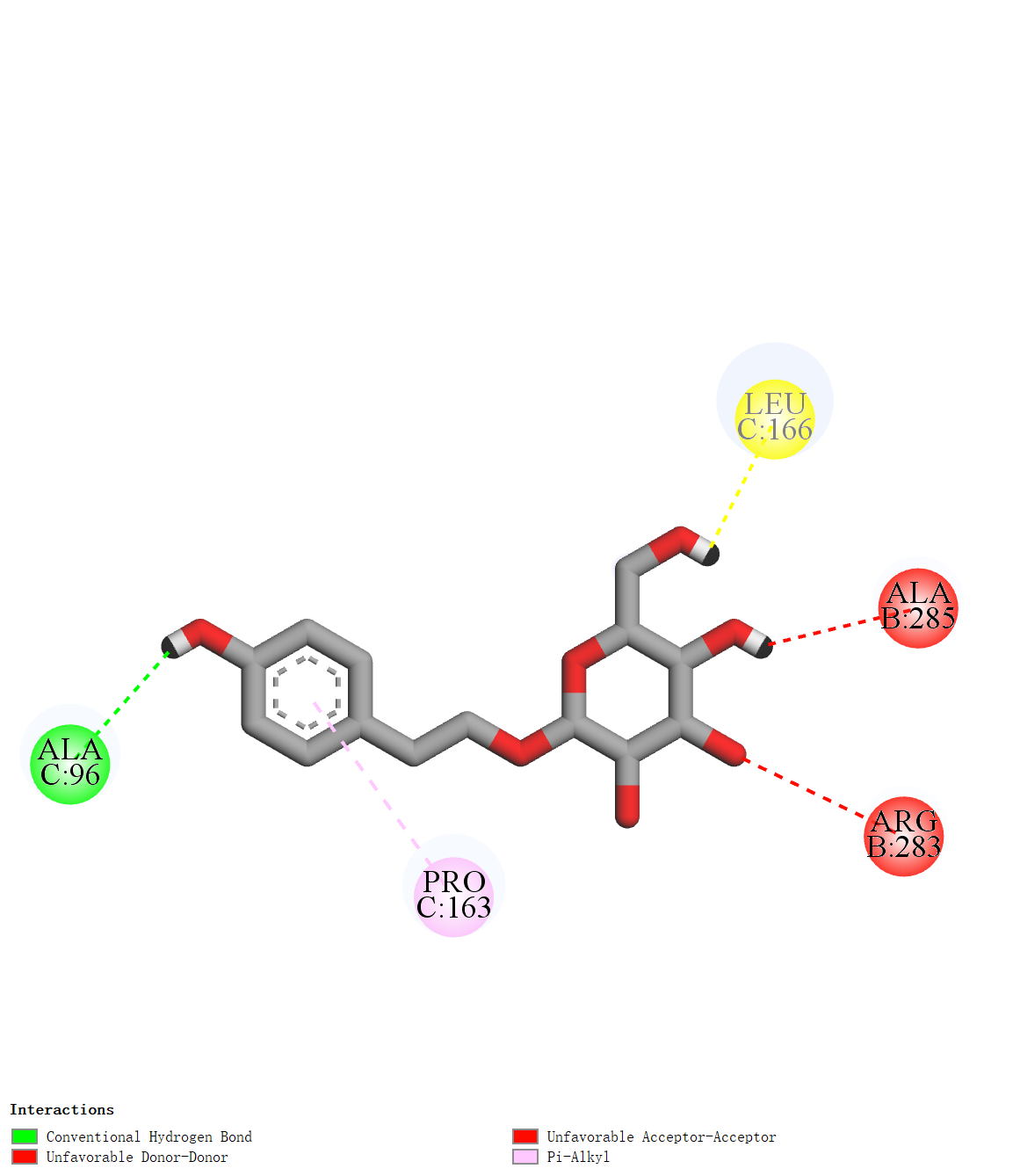

Supplement: Supplementary file 1 [file DataSheet1.ZIP › Supplementary document/Molecular docking/Figure/ECHS1-3.png]

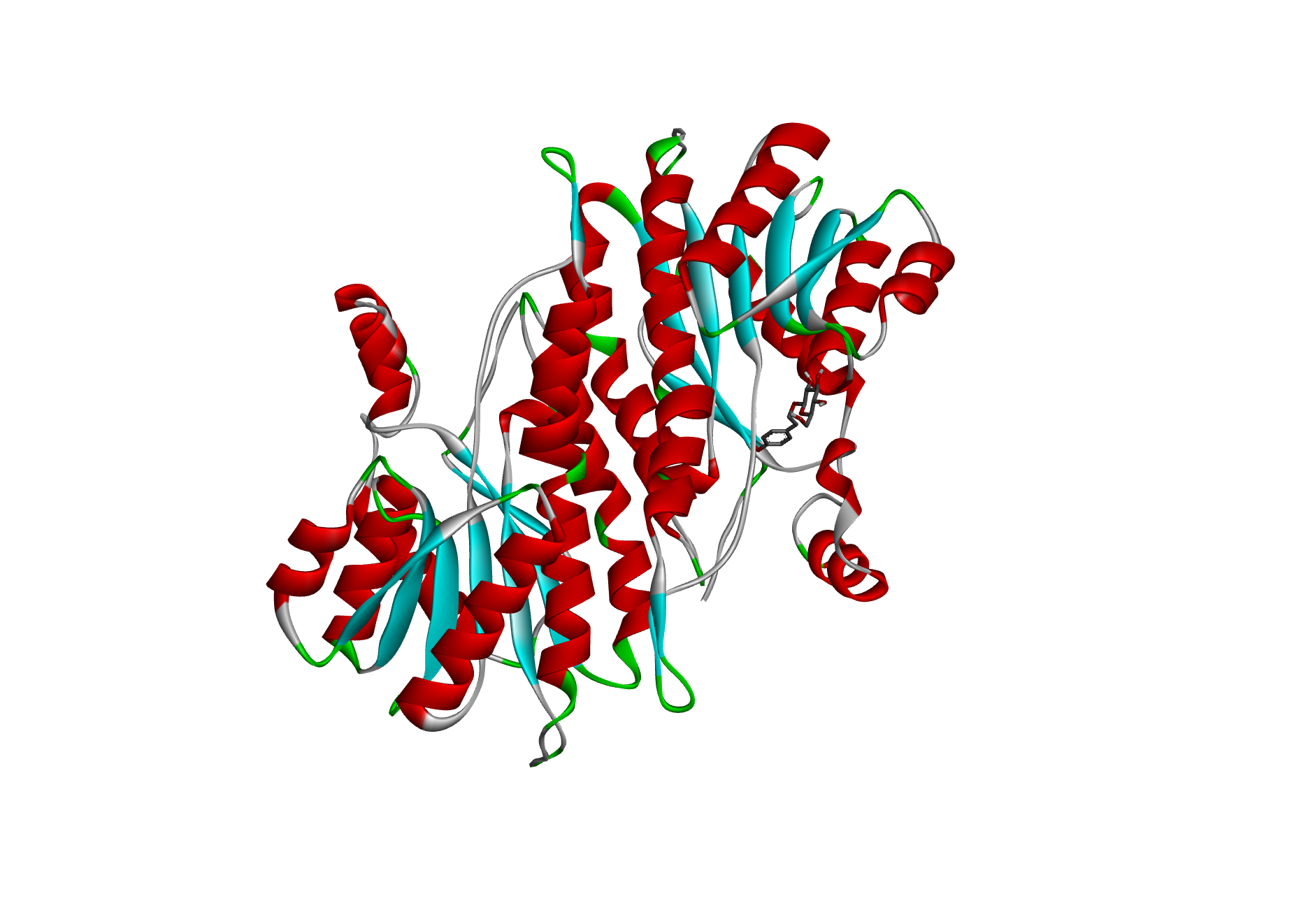

Supplement: Supplementary file 1 [file DataSheet1.ZIP › Supplementary document/Molecular docking/Figure/HSD17B10-1.png]

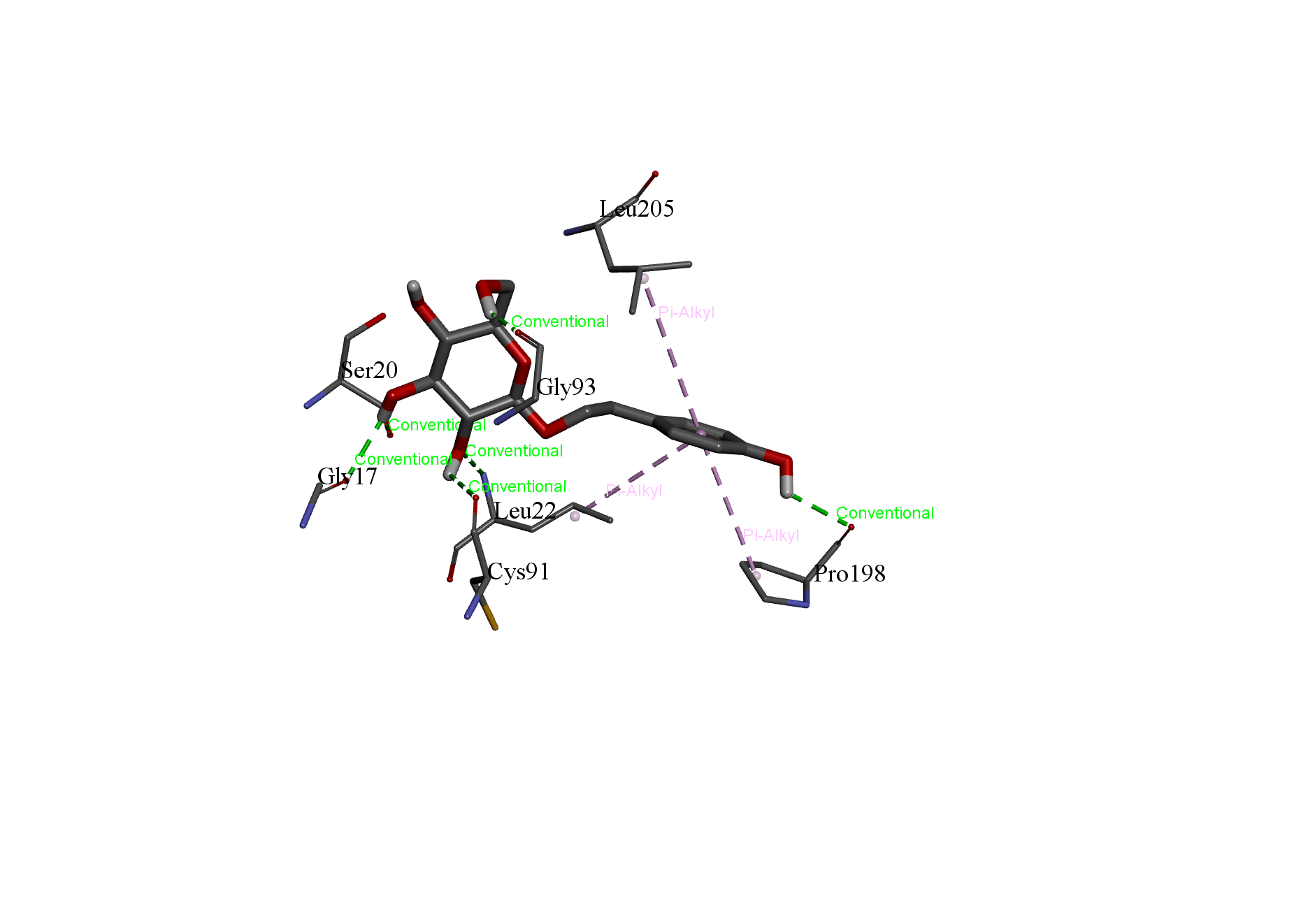

Supplement: Supplementary file 1 [file DataSheet1.ZIP › Supplementary document/Molecular docking/Figure/HSD17B10-2.png]

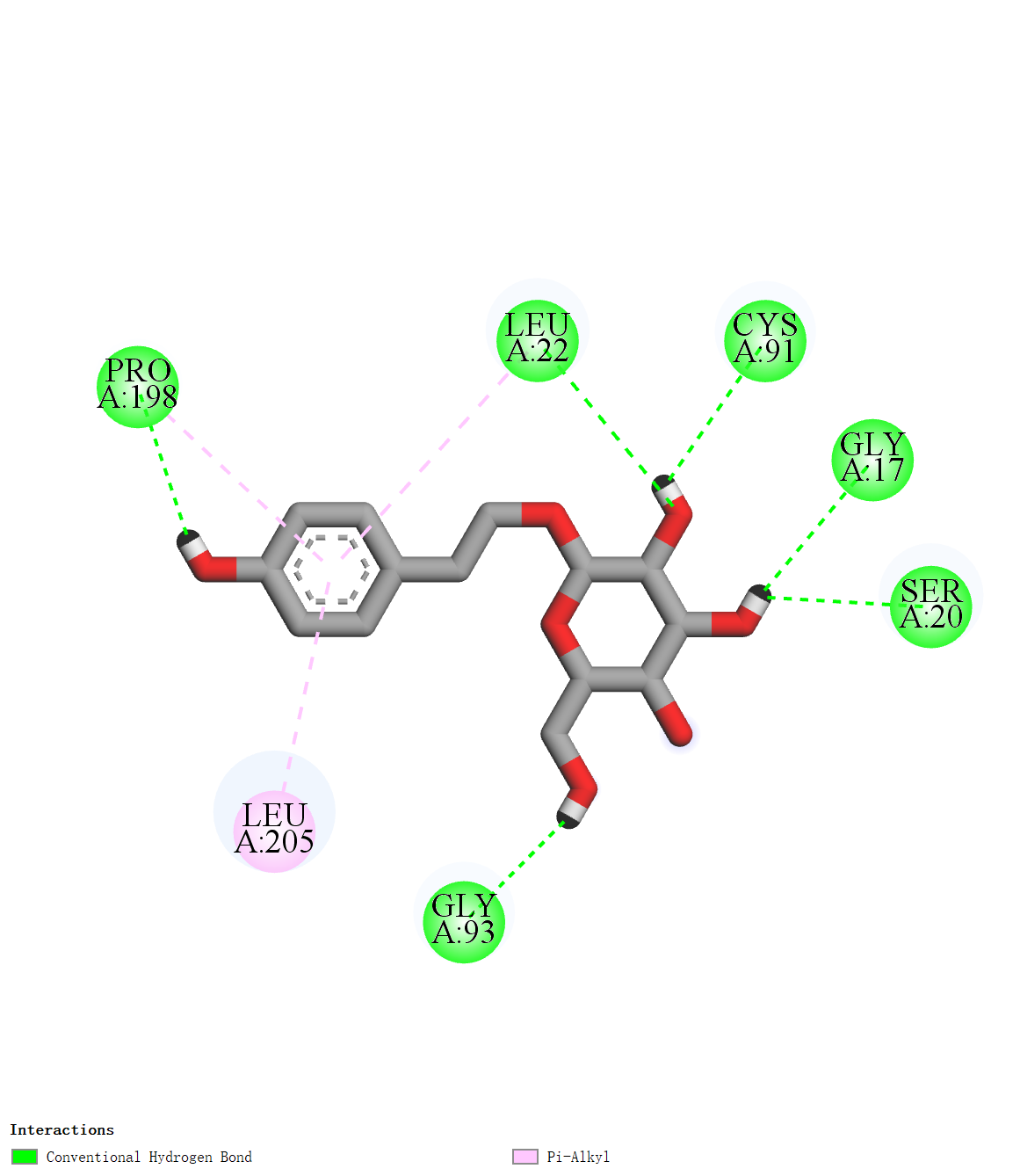

Supplement: Supplementary file 1 [file DataSheet1.ZIP › Supplementary document/Molecular docking/Figure/HSD17B10-3.png]

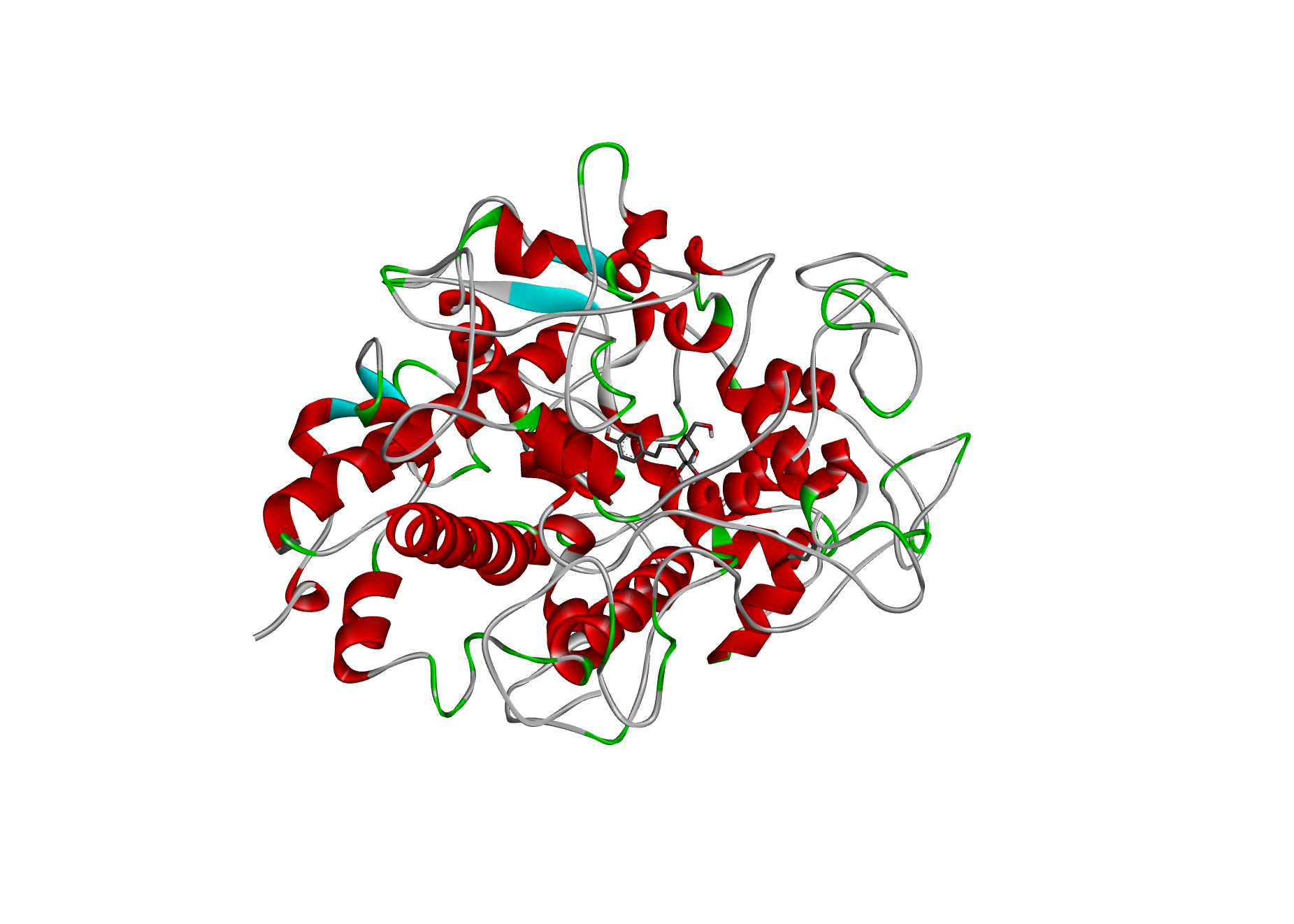

Supplement: Supplementary file 1 [file DataSheet1.ZIP › Supplementary document/Molecular docking/Figure/MPO-1.png]

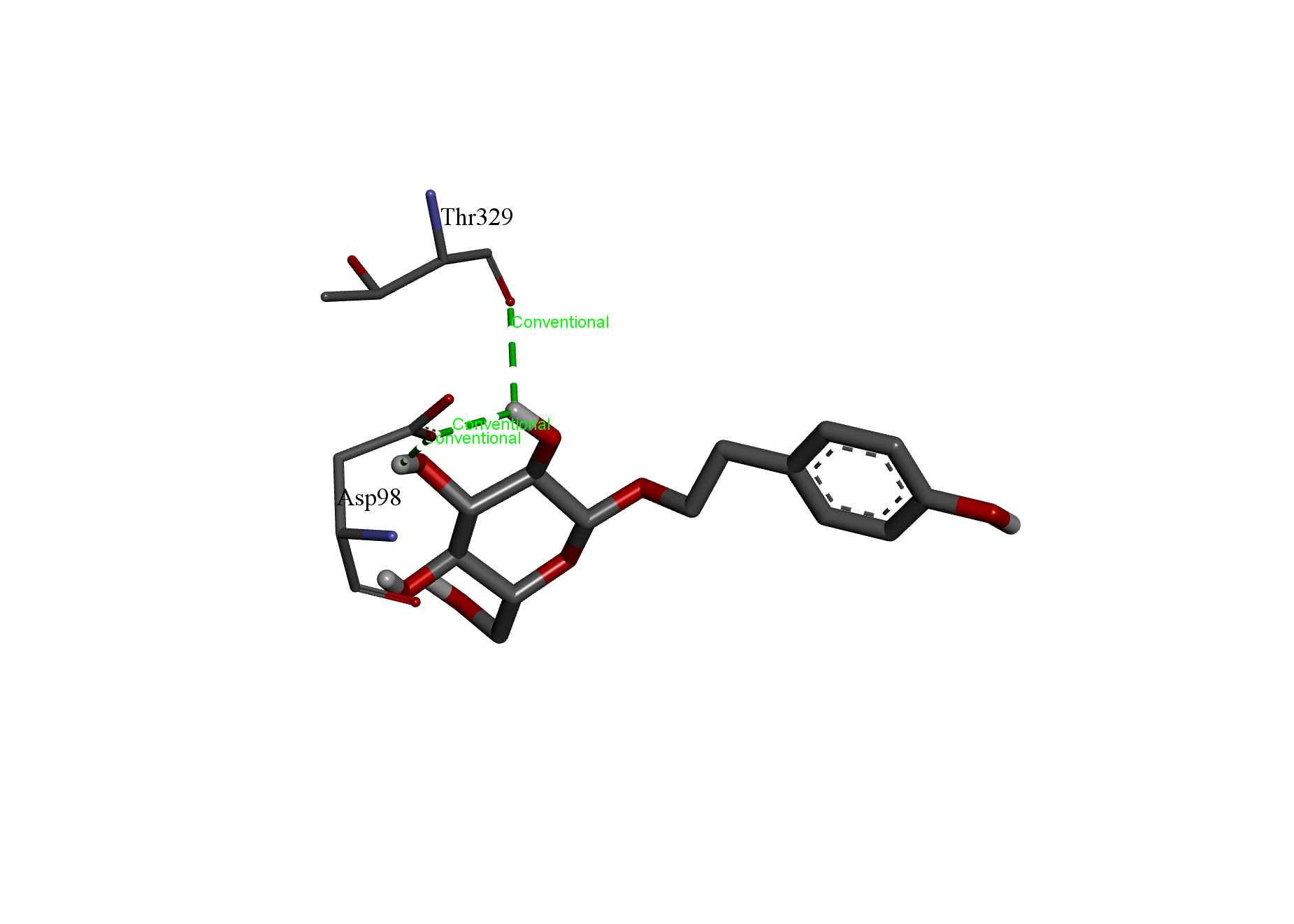

Supplement: Supplementary file 1 [file DataSheet1.ZIP › Supplementary document/Molecular docking/Figure/MPO-2.png]

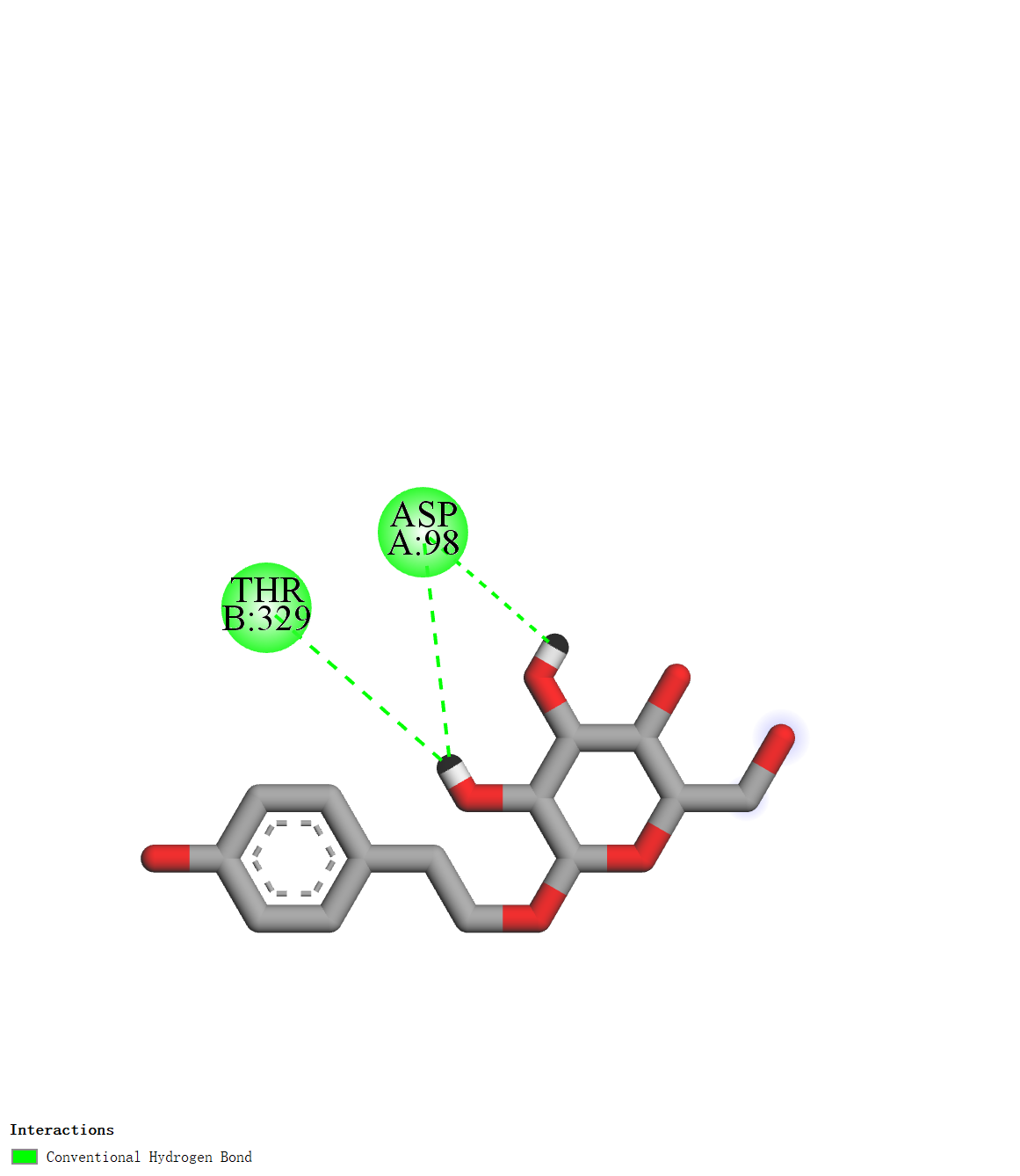

Supplement: Supplementary file 1 [file DataSheet1.ZIP › Supplementary document/Molecular docking/Figure/MPO-3.png]

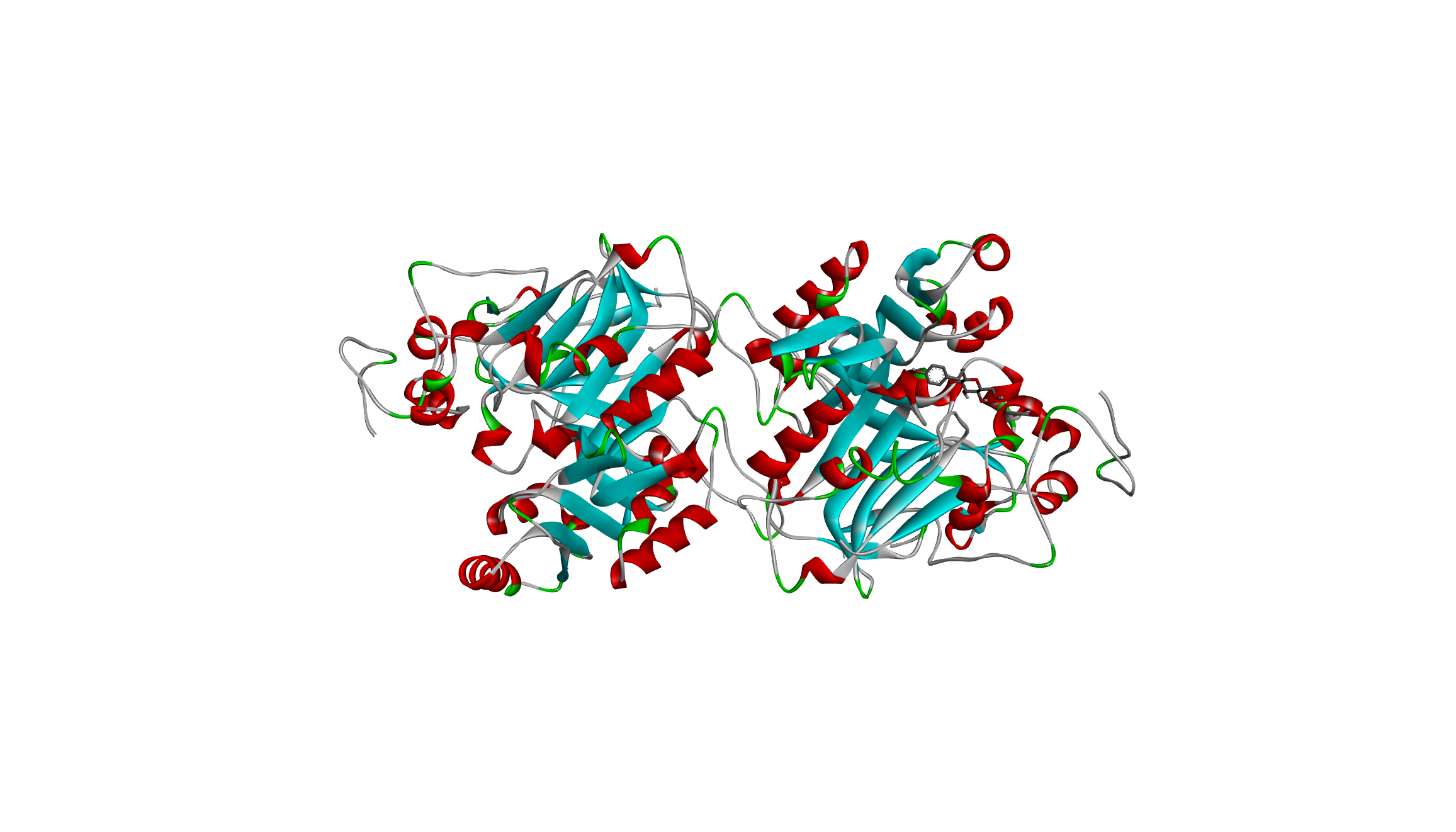

Supplement: Supplementary file 1 [file DataSheet1.ZIP › Supplementary document/Molecular docking/Figure/TYR-1.png]

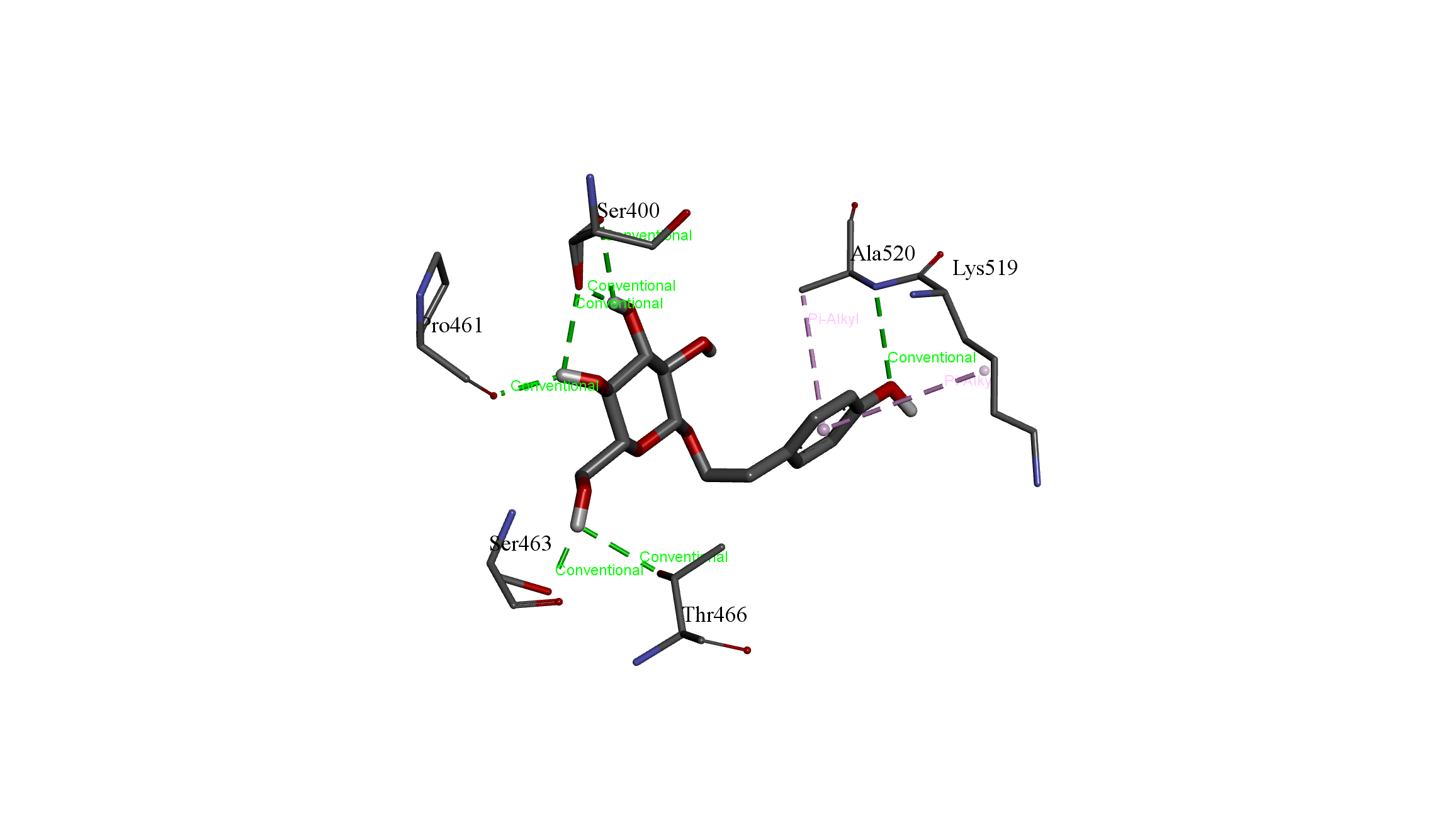

Supplement: Supplementary file 1 [file DataSheet1.ZIP › Supplementary document/Molecular docking/Figure/TYR-2.png]

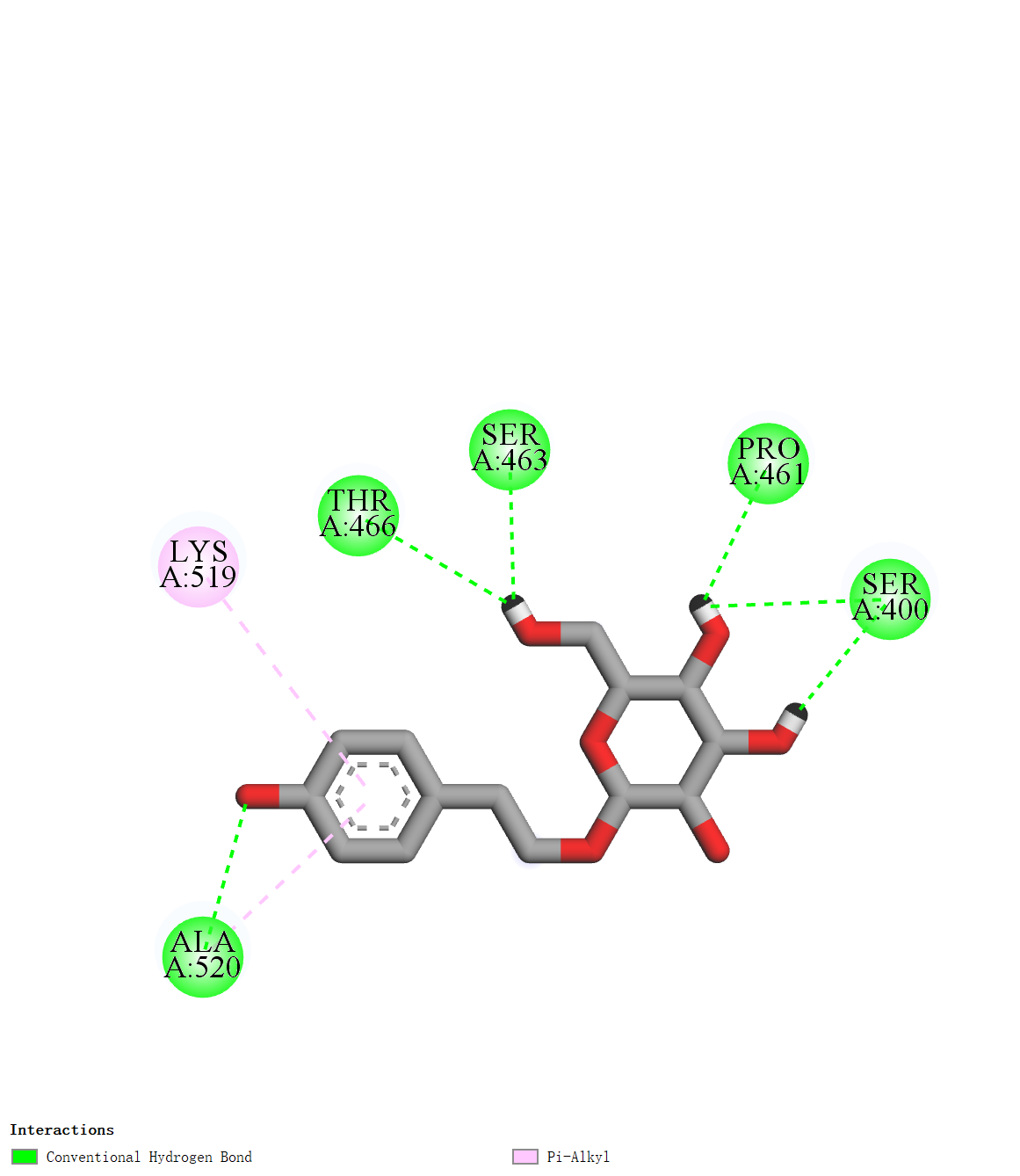

Supplement: Supplementary file 1 [file DataSheet1.ZIP › Supplementary document/Molecular docking/Figure/TYR-3.png]
